# A murine model of atopic dermatitis can be generated by painting the dorsal skin with hapten twice 14 days apart

**DOI:** 10.1038/s41598-018-24363-6

**Published:** 2018-04-16

**Authors:** Ayaka Kitamura, Ryohei Takata, Shin Aizawa, Hajime Watanabe, Tadashi Wada

**Affiliations:** 1 Nucleic Acid Regulation (Yoshindo) Joint Research Laboratory, Department of Biotechnology, Osaka University, Osaka, Japan; 2 Bioenvironmental Science, Department of Biotechnology, Osaka University, Osaka, Japan; 3 Department of Research and Development, Yoshindo Inc., Toyama, Japan; 4 Department of Functional Morphology, Nihon University School of Medicine, Tokyo, Japan

## Abstract

Drug development involves pharmacometric experiments in animals. Such experiments should limit animal pain and stress. Conventional murine models of atopic dermatitis (AD) used in drug development are generated by weekly painting of hapten on dorsal skin for 5 weeks. The present study aimed to develop a protocol that involves less animal distress. The experiments focused on serum total IgE levels, which are a marker of AD. The conventional protocol induced ever rising IgE levels. Experiments with extended intervals between sensitizations showed that IgE peaked ~5 days after the second sensitization, after which it returned to the control level within 12–19 days. An additional third sensitization on day 28 further increased the serum IgE level. In the 4–5 days after the second sensitization, the dorsal skin exhibited typical AD-like lesions with edema, scabs, epithelial-cell hypertrophy, marked mast-cell and lymphocyte infiltration of dermis, and increased IL-4, IL-6, IL-10, IL-1β, IL-17A, IFN-γ and TNF-α expression. Thus, two 2,4-dinitrofluorobenzene sensitizations yield a murine AD model in less than 20 days. This study shows that animal model protocols used in drug development can be fine-tuned so that they remain effective yet cause animals less stress and pain.

## Introduction

During drug discovery, it is necessary to use animals for various experiments, including for evaluating the efficacy and safety of the candidate drug. However, we should minimize the number of animals used in such experiments and reduce their stress and pain as much as possible. This can best be achieved using simple standard methods that generate reliable results while imposing the smallest possible burden on the animals. The development of such methods is thus an important research priority.

There are many patients with atopic dermatitis (AD) all over the world^1,2^. AD is a serious skin disease that is characterized clinically by eczema and itching^3,4^. Its key immunological feature is high levels of serum total IgE^5,6^. These high IgE levels are induced by some leukocytes; including T helper type 2 (Th2) cells that are specific for allergens such as certain foods and insect proteins. When these cells in the circulation encounter these allergens, they produce inflammatory cytokines such as interleukin (IL)-4. In turn, Th2 cells stimulate B cells to produce IgE in spleen^7–10^, after they have been presented to the antigen by leukocytes. The serum IgE then activates a variety of immune cells that bear IgE receptors on their cell surface, including mast cells, basophils, and eosinophils. The binding of antigen by receptor-bound IgE induces the cells to secrete inflammatory cytokines, chemokines, histamine, and leukotriene. These molecules in turn promote other inflammatory reactions that eventually lead to further exacerbation of the clinical manifestations of AD^11^. As a result of this immune cascade, serum total IgE concentrations generally correlate positively with AD symptoms^12^, although further research on this relationship is needed.

Effective therapies for AD are still lacking because only its symptoms, not the cause of the disease, are addressed by current treatments. Consequently, there is considerable research into the development of potential drugs that involves animal models of AD^13^. In particular, AD phenotypes can be reproducibly induced by painting haptens, including 2,4-dinitrofluorobenzene (DNFB) and 2,4-dinitrochlorobenzene (DNCB) on the dorsal skin and ears of mice. More than 130 papers have described a protocol that generates a murine model of AD with DNFB or DNCB painting (Supplementary Fig. S1). These models always involve multiple hapten painting sessions, many of which activate the immune response shift from T helper type1 (Th1) to Th2^14–16^, a signature of AD, and serum total IgE concentrations increase as the painting frequency increases^17^. Other animal models of AD are generated using mite extracts and other allergens, most of which also involve repeated applications^18–20^. Thus, the generation of the current animal models of AD requires repeated painting sessions, each of which inevitably induces stress and pain in the animals.

Recent progress in this field has clarified the mechanism of how some leucocytes contribute to the pathogenesis and exacerbation of dermatitis. Immune cell specific depletion using genetic engineering has enabled exploration of the function of immune cells more accurately than that using antibody-mediated depletion strategy, which induces long-standing effects^21^. Transgenic mice, which contain less basophils, demonstrated that basophils play a pivotal role in Th2 cell differentiation and probably IgE production^22^.

During our research into therapeutic drugs for AD, we sought to develop a murine model of AD that causes the least possible pain and stress in the mice. In the present study, five different protocols were performed. The protocols differed in terms of the number of times dorsal painting with hapten and the interval between the painting sessions. The information obtained from these experiments eventually led to a protocol that yielded good AD while inducing relatively little pain and stress in the mice. To our knowledge, studies searching for such relatively painless and stressless protocols have not yet been published.

## Results

### Protocol 1: Conventional DNFB sensitization once weekly for 5 weeks

**Figure 1:**
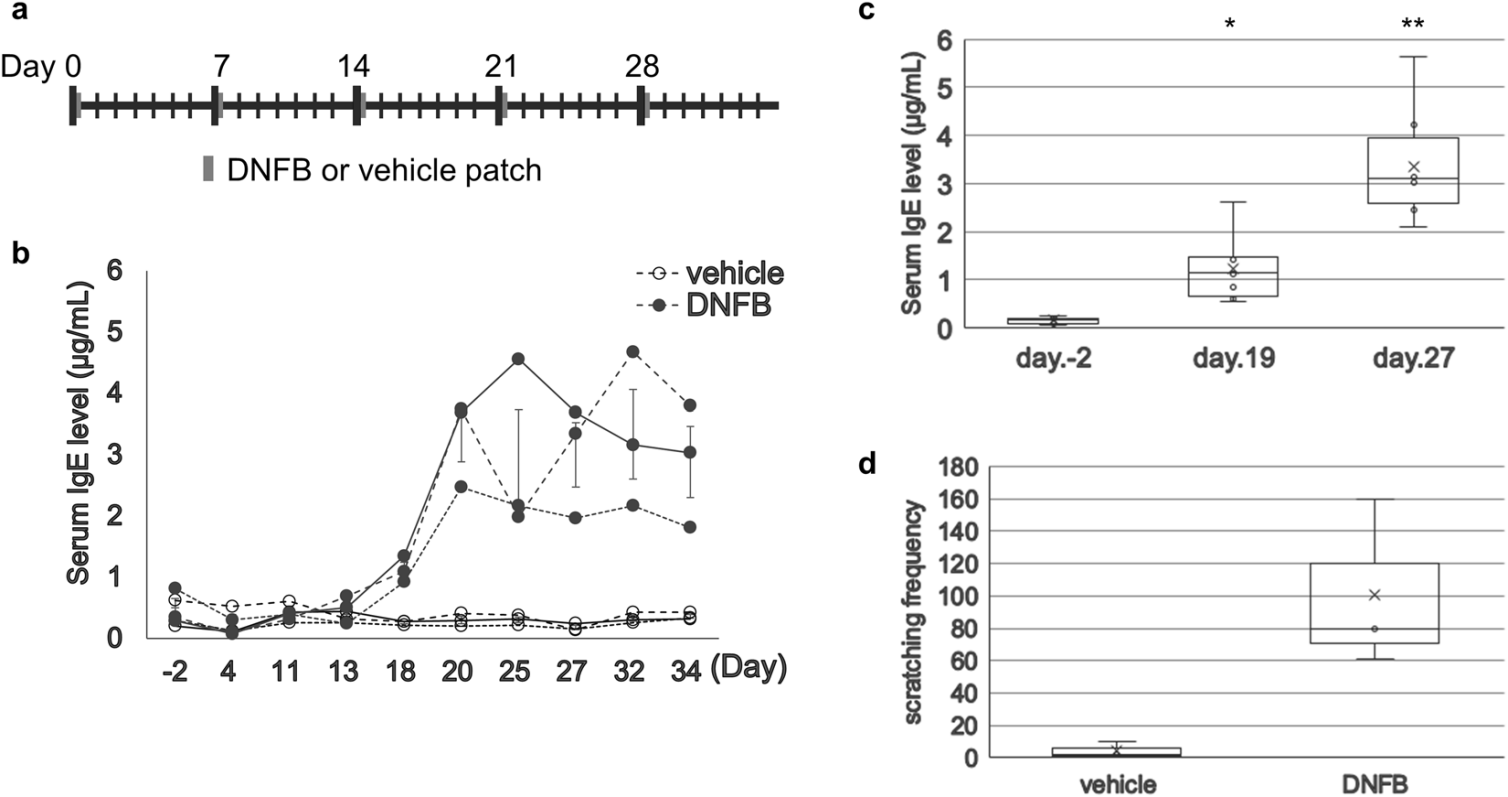
Change in serum total IgE levels of mice sensitized with DNFB according to Protocol 1. (**a**) Protocol 1 is a conventional protocol for hapten-induced dermatitis. It involves five DNFB sensitizations at weekly intervals. (**b** and **c**) The dorsal skin of BALB/c mice was painted with 100 µL of 0.15% DNFB in vehicle [acetone/olive oil (3:1)] (black circle of b, n = 3 and c, n = 8) or 100 µL of vehicle (white circle of b, n = 3) every week for 4 weeks. Blood samples were collected from the legs at the indicated time points. The serum IgE concentrations were measured by sandwich ELISA. (**b**) and (**c**) indicates different mice. The individual data of the three mice that underwent painting with DNFB and vehicle, respectively, are shown (**b**). (**d**) The frequency of scratching behavior of three mice per group is indicated. The movie was recorded for 15 min after 5^th^ DNFB treatment. (**c**,**d**) The distributions of IgE and scratching frequency are represented as a box plot together with the medium and inter quarter range (IQR). *P < 0.05 and **P < 0.01, versus IgE value of the pre-treatment. The x-axis is not drawn to scale.

Current murine models of AD are generated by repeated painting of DNFB or DNCB on the dorsal skin and ears (Supplementary Fig. S1). We started the present study with Protocol 1, which is a commonly used method for generating animal models of AD with a hapten. It involves painting the dorsal skin of mice with DNFB every week for 5 weeks (Fig. 1a). Thus, it constitutes five painting sessions. We painted three mice in this way. As a control, we painted three additional mice with vehicle on the same days. We collected blood 2 days before starting painting and then on the days indicated in Fig. 1b. The serum total IgE levels were determined by ELISA. The IgE levels of the individual DNFB-painted mice are shown in Fig. 1b. The IgE levels began to increase after the third painting session. While there was a tendency to peak slightly 20–32 days after the first DNFB sensitization, the IgE levels remained continuously high until the end of the 34 day observation period (Fig. 1b). The mice differed in the height of their peaks and when they occurred. This may reflect individual differences or an irrelevant condition. Since the vehicle-painted mice exhibited low IgE levels throughout the 5 week study period (Fig. 1b), the increased serum IgE levels in the DNFB-painted mice were induced by DNFB sensitization. We collected blood from mice 2 days before and 19 and 27 days after 1^st^ sensitization to measure serum IgE levels (Fig. 1c). The IgE ratios of post-sensitized mice were significantly higher than those before sensitization (*P* = 0.03, day 19 and *P* = 1.0 × 10^−7^, day 27).

Since itching is a fundamental atopic dermatitis symptom, we counted the number of scratching events of DNFB-painted and vehicle-painted mice, as described in ref.^23^. The scratching behaviors of three mice per group were recorded for 15 min after the 5^th^ DNFB painting on day 28. DNFB-painted mice showed more frequent scratching behavior than vehicle-painted mice (Fig. 1d) and long-lasting itching when mice were observed twice a week during the experiment (Supplementary Fig. S2).

### Protocols 2–4: DNFB sensitization once weekly for 3 weeks, then 9 days later, then 12, 19, or 26 days later

Protocols 2–4 were inspired by previously reported allergen exposure experiments. Talay, O. *et al*. showed that serum total IgE concentrations peaked ~15 days after *Nippostrongylus brasiliensis* infection, after which the IgE levels waned steadily until around day 24^24^. Yamamoto, T. *et al*. and Sokol, C. L. *et al*.^25,26^ reported increased concentrations of IgE 14 days after intravenously ovalbumin (OVA) challenge following OVA pulsed basophil injection through the tail vein and subcutaneous immunization with papain, a protease allergen, respectively. These immunizations induced Th2 cell differentiation, which was promoted by IL-4 stimulation, and led to atopic dermatitis in lesional skin. Perrigoue, J. G. *et al*. showed that Th2 cytokine-dependent resistin-like molecule-β (RELMβ) expression was higher on days 12–18 after *Trichuris. muris* infection^27^. The presence of Th2 cells in peripheral tissue is a clinical feature of atopic dermatitis.

These observations, together with the Protocol 1 findings, indicate that, when constructing a protocol for generating an animal model of AD that involves fewer painting sessions than conventional methods, several aspects must be considered. First, it is clear that one DNFB painting session is not sufficient to induce AD-like reactions. Second, infection with helminth parasites, including *N. brasiliensis* and *T. muris*, suggested that it is possible that, if sufficient time elapses after the first sensitization, a second sensitization may efficiently generate AD-like reactions. Third, the Protocol 1 findings and allergen immunization experiments indicated that the first and second sensitizations should be separated by more than 7 days. This led to experiments to determine the optimal duration between the first and second sensitizations that allows IgE levels to peak and then return to the control level before the third sensitization is administered.

**Figure 2:**
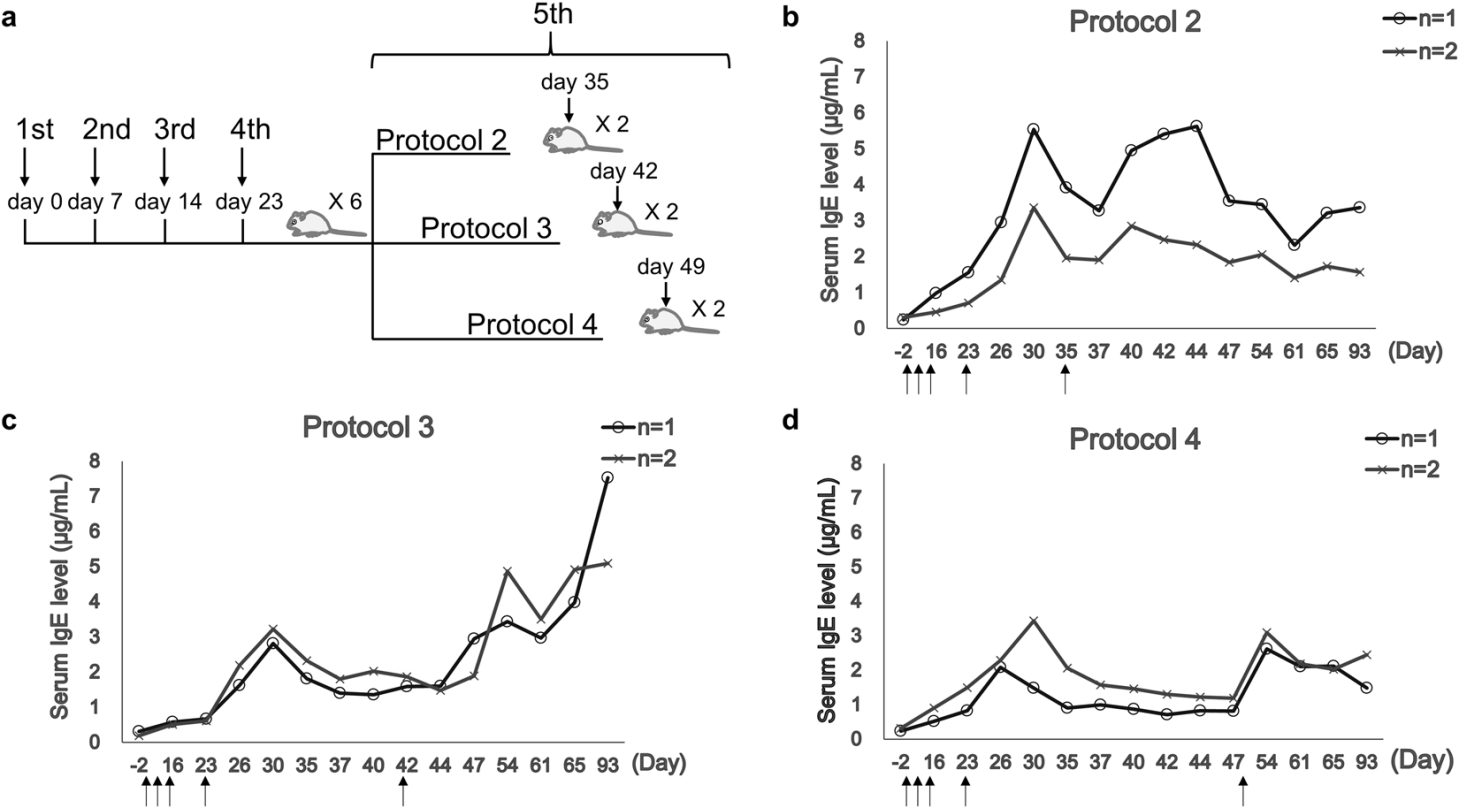
Change in serum total IgE levels of mice sensitized with DNFB according to Protocol 2, 3, or 4. (**a**) Six mice underwent weekly DNFB painting sessions (100 µL of 0.15% DNFB) for 3 weeks. They were then painted a fourth time 9 days later (day 23) and divided into three groups of two. Protocols 2, 3, and 4, respectively, involved a fifth painting session 12, 19, or 26 days after the fourth session (on days 35, 42, and 49, respectively). (**b**) Protocol 2. (**c**) Protocol 3. (**d**) Protocol 4. Blood samples were collected from the legs at the indicated time points. The serum IgE concentrations were measured by sandwich ELISA. The x-axis is not drawn to scale. The arrows indicate DNFB painting.

Thus, an experiment with Protocols 2, 3, and 4 was performed. Six mice underwent weekly painting sessions for 3 weeks, as in Protocol 1. They were then painted for the fourth time 9 days later (day 23). The mice were then divided into three groups of two. The mice in Protocols 2, 3, and 4, respectively, underwent a fifth painting session 12, 19, or 26 days after the fourth session (on days 35, 42, and 49, respectively) (Fig. 2a).

When the interval between the fourth and fifth painting sessions was 12 days (Protocol 2), the IgE levels peaked **7 days** after the fourth session and then started to wane. The fifth session was then performed: it rapidly induced a second peak **5–9 days** later. The IgE levels then started to wane (Fig. 2b).

When the interval between the fourth and fifth painting sessions was 19 days (Protocol 3), the IgE levels peaked **7 days** after the fourth session and then waned to a steady state. The fifth sensitization was then administered: the IgE levels peaked **12 days** later. After the latter peak, the IgE levels waned slightly before spontaneously rising again. As a result, the highest IgE levels in the Protocol 3 mice were on the last day of observation (day 93) (Fig. 2c).

When the interval between the fourth and fifth painting sessions was 26 days (Protocol 4), the IgE levels peaked **3–7 days** after the fourth session and waned to a steady state. The fifth sensitization was then administered, and the IgE levels peaked **5 days** later, followed by slight waning (Fig. 2d).

These results indicate that the most optimal interval between painting sessions is >12 days and <19 days. In addition, it seems that IgE concentrations start rising **on average ~5 days** after DNFB sensitization.

### Protocol 5: Three DNFB sensitizations, each 2 weeks apart

**Figure 3:**
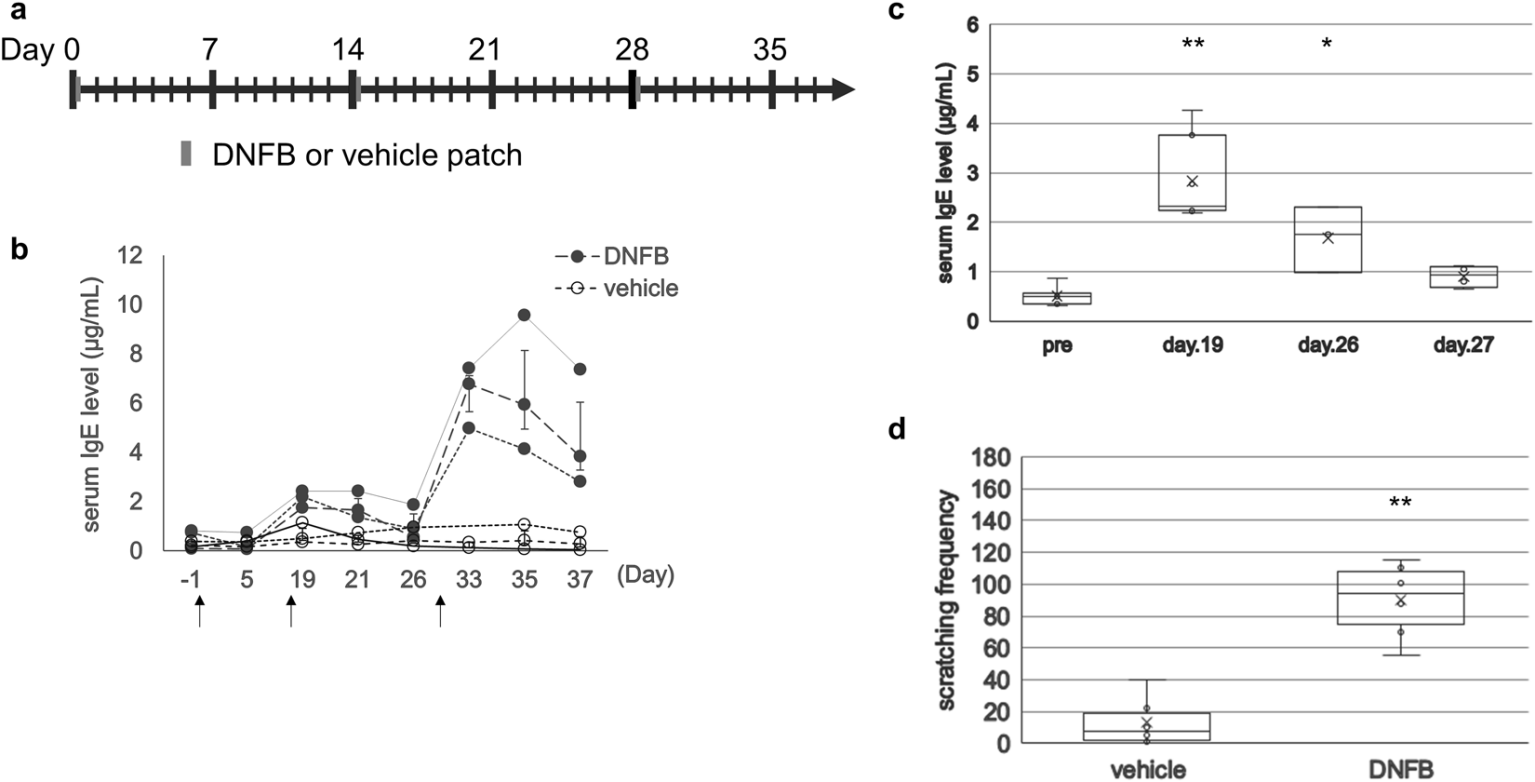
Change in serum total IgE levels of mice sensitized with DNFB according to Protocol 5. (**a**) Protocol 5 involves three DNFB sensitizations at 14 day intervals. (**b** and **c**) Mice were painted with 100 µL of 0.15% DNFB in vehicle [acetone/olive oil (3:1)] (black circle of b, n = 3 and c, n = 7, pre and day 19, n = 3, day 26 and n = 4, day 27) or 100 µL of vehicle only (white circle of b, n = 3). (**b**) and (**c**) indicates different mice. Blood samples were collected from the legs at the indicated time points. The serum IgE concentrations were measured by sandwich ELISA. IgE values of the individual mice are presented as a line graph (three mice per group) (**b**) and as a box-plot with the median and IQR of 3–7 mice (**c**). *P < 0.05 and **P < 0.01, versus IgE values of pre-treatments. The x-axis is not drawn to scale. The arrows indicate DNFB painting. (**d**) The frequency of scratching behavior was observed 2 h after 2^nd^ DNFB treatment. Data are presented as a box-plot with the median and IQR of six mice. **P < 0.01, versus vehicle-treated mice.

These results led us to Protocol 5, which involved three DNFB sensitizations at 14 day intervals (Fig. 3a). In three mice treated with Protocol 5, the IgE levels were low 5 days after the first sensitization. This indicates that a single sensitization did not significantly affect total serum IgE concentrations. After the second sensitization, however, the IgE levels peaked 5 days later. Thus, the second sensitization stimulated IgE production in all of the mice (Fig. 3b). The IgE levels then dropped gradually, at which point the third sensitization was applied (day 28). This led to a large peak 5 days later (Fig. 3b). Thereafter, the total IgE concentration started declining again. By contrast, vehicle-treated mice did not exhibit any marked increases in IgE levels (Fig. 3b). This pattern of IgE responses was confirmed (Fig. 3c, *P* = 3.4 × 10^−6^, day 19 and *P* = 0.04, day 26).

Since scratching behaviour is one of the most common symptoms of AD, we investigated whether two DNFB sensitizations at 14 day intervals induced scratching behavior to mice. DNFB-painted mice showed frequent scratching behavior 2 hr after the 2^nd^ DNFB painting (Fig. 3d) similar to that observed after the conventional one (Fig. 1d; Protocol 1). Protocol 5 did not induce so long-lasting scratching behaviour to DNFB-painted mice as Protocol 1 did.

### Generation of a murine model system of AD

**Figure 4:**
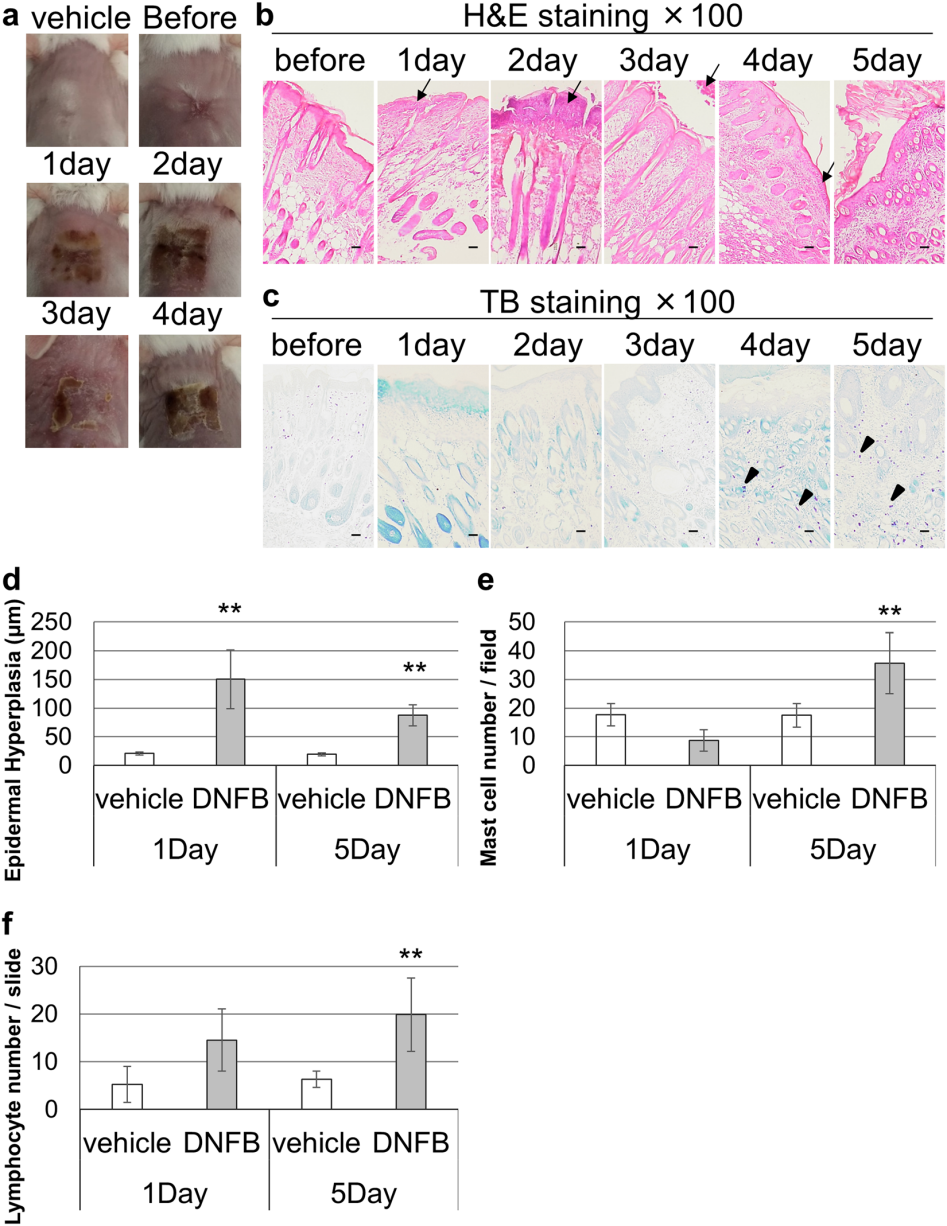
Gross morphology and histology of the dorsal skin of mice that underwent Protocol 5. (**a**) Photos of the dorsal skin were taken before and 1, 2, 3, and 4 days after the second DNFB painting session. For comparison, the dorsal skin of a control mouse that was painted with vehicle was photographed 4 days after the second vehicle application. (**b** and **c**) Hematoxylin and eosin staining (**b**) and toluidine blue staining (**c**) of the dorsal skin samples taken before and 1, 2, 3, 4 and 5 days after the second DNFB painting session. The arrows indicate skin lesions, such as oedema and lymphocytes, and the arrowheads indicate mast cells. Magnification, ×100; scale bar = 100 μm. (**d**) Epidermal thickness was measured from H&E stained images (n = 5, Mean and s.d.). (**e**) Infiltration of mast cells into dermal and subcutaneous tissue was observed and counted (n = 4, DNFB 1 day, n = 5, vehicle and n = 6, DNFB 5 day, Mean and s.d.). (**f**) Lymphocyte accumulation in subcutaneous tissue was observed and counted (n = 5, Mean and s.d.). **P < 0.01, versus vehicle-treated mice.

To determine whether two sensitizations would be sufficient to induce AD-like skin lesions, we photographed the dorsal skin of mice that underwent Protocol 5 before and every day after the second sensitization for 4 days. Skin lesions appeared 1 day after the second sensitization (Fig. 4a). The lesions had signs of severe skin damage, including redness, edema, and scabs. The scabs were particularly apparent between days 2 and 4 (Fig. 4a).

Hematoxylin and eosin (H&E) staining of the DNFB-painted skin revealed initiation of epidermal hypertrophy on day 1 and clear inflammation, characterized by exacerbated intracellular oedema of the epidermis, on day 2. During days 2–3, the stratum corneum thickened and partially disrupted (Fig. 4b). The epidermal thickness on days 1 and 5 was measured quantitatively and epidermal hypertrophy was observed and compared to the vehicle-treated mice skin (Fig. 4d, *P* = 2.6 × 10^–5^, 1 day DNFB and *P* = 0.01, 5 day DNFB and Supplementary Table S1 and Fig. S3). Thus, the second sensitization induced AD-like signs. Stratum corneum regeneration seemed to start on day 4 (Fig. 4b). H&E staining and Toluidine blue (TB) staining showed that the dermis and subcutaneous tissue were extensively infiltrated with leukocytes, including lymphocytes and mast cells, on day 4 (Fig. 4b,c, arrow and arrowhead, respectively). On days 4 and 5, the skin lesions contained many mast cells that looked larger in size than on days 1–3 (Fig. 4c). On day 5, mast cells infiltrated the lesional skin, while they were absent on day 1 (Fig. 4e, *P* < 0.01 and Supplementary Table S2 and Fig. S4). Lymphocytes accumulated to higher levels in lesional skin on day 5 than in vehicle-treated skin (Fig. 4f, *P* < 0.01 and Supplementary Table S3 and Fig. S5). These results suggest that two painting sessions are sufficient to induce AD-like skin phenotypes if they are 14 days apart.

**Figure 5:**
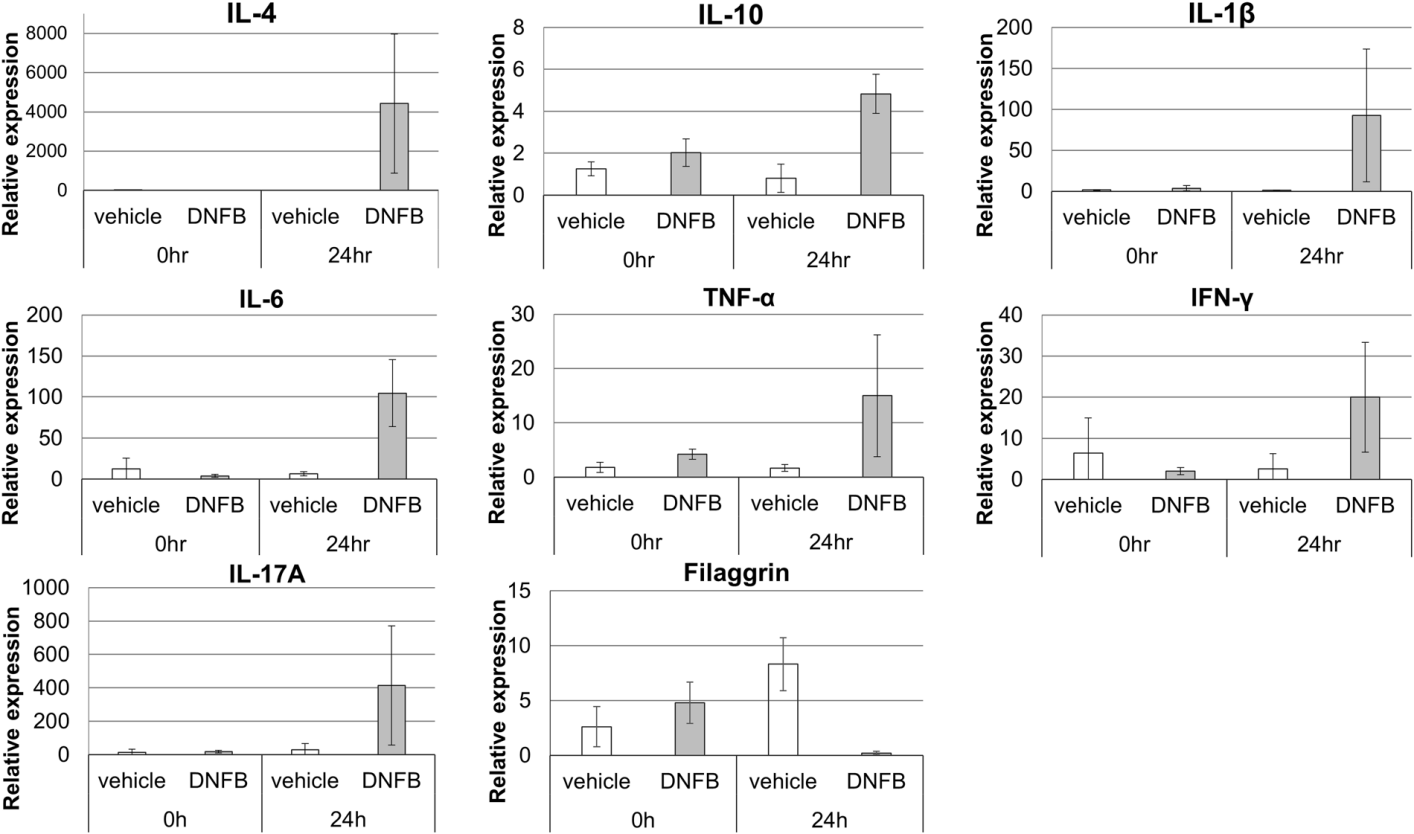
Expression of inflammatory cytokines and AD-related gene in the painted skin before and 24 h after the second painting session in Protocol 5. Total RNA was extracted from the dorsal skin of the Protocol 5-treated mice before (0 h) and 24 h after the second DNFB painting sessions. Quantitative PCR was performed to determine the mRNA expression of IL-4, IL-6, IL-10, IL-17A, IL-1β, IFN-γ, filaggrin, and TNF-α after second painting. Mean and s.d. of three mice per group.

To confirm these findings, the dorsal skin samples taken from Protocol 5-treated mice and vehicle-treated mice just before (0 h) and 24 h after the second painting sessions were subjected to quantitative PCR and the expression of inflammatory cytokines and AD-related genes was measured. As shown in Fig. 5, the DNFB-painted dorsal skin of the mice exhibited IL-4 expression 24 h after the second painting sessions. IL-4 expression was not observed after the first painting session and was never observed in the vehicle-treated mice (Fig. 5 and Supplementary Fig. S6). Similarly, the DNFB-painted dorsal skin of the mice exhibited strong IL-6, IL-10 and TNF-α mRNA expression after the second and third painting sessions (Fig. 5 and Supplementary Fig. S6), although the reason why cytokine expression induced by the second session was similar to that after the third session is unclear. Moreover, the expression levels of IL-1β, IFN-γ, and IL-17A mRNA were remarkably increased at 24 h after the 2^nd^ DNFB painting (Fig. 5). Interestingly, filaggrin gene expression was strongly decreased (Fig. 5).

These results indicate strongly that two DNFB sensitizations 14 days apart are sufficient to induce AD signs and symptoms. Thus, this protocol, which uses two sensitizations rather than the five that are conventionally used, reliably and effectively generates a murine model of AD. Not only does this model place a lower burden on the mice in terms of pain and stress than conventional methods; it is simpler and the mouse model is generated faster.

## Discussion

It is important to ensure that laboratory experiments involving mice reduce the pain and stress of the animals as much as possible. In the present study, we searched for a protocol that can effectively and reliably generate a mouse model of AD with much less stress and pain for the mice than conventional methods. This new protocol involves painting the dorsal skin of the mice with DNFB twice at 14 day intervals. This approach effectively increases serum total IgE levels and scratching behaviour of mice (Fig. 3), induces AD-like skin lesions (Fig. 4), elevates the skin levels of cytokines that are known to participate in AD^13,28–33^, and decreases the skin level of the AD-related filaggrin gene^34^ (Fig. 5). In addition, further increases in the serum IgE level were observed 5 days after the third sensitization on day 28. The scratching behaviour of Protocol 5-treated mice was not sustained in longer term than that of Protocol 1 (Supplementary Fig. S2), although Protocol 5 induced similar IgE increase to Protocol 1 (Fig. 1c, day 27 and Fig. 3c, day 19). To develop this protocol, we used a conventional method (Protocol 1) and three variations of this protocol (Protocols 2–4) to identify the best time points for the sensitizations. These experiments showed that IgE levels started to rise **~5 days** after sensitization following an earlier sensitization (Figs 1–3) and that sensitizations should be separated by >12 days and <19 days to generate the best response. Indeed, a second sensitization 14 days after the first effectively and reliably induced strong IgE peaks and AD-like signs and skin cytokine responses.

Many studies show that weekly sensitization with hapten, insect extracts, or foods over a period of more than 1 month (*e.g*., Protocol 1) yields continuously rising IgE concentrations^35^ (Figs 1, 2, and Supplementary Fig. S1). By contrast, Protocol 5 induces IgE peaks that emerge **~5 days** after the second sensitization; the IgE levels then start to wane and reach a steady state somewhere between 9 and 14 days later (Fig. 3). It is likely that Protocol 1-like methods do not induce IgE peaks because it takes more than 7 days for the rising IgE concentrations to peak. Recent studies reported that basophils mediate Th2 immune responses induced by the protease allergen, OVA, and helminth infection^25–27^. Basophils present the antigen to naïve T cells and promote differentiation into Th2 cells^36,37^. Basophil appearance and Th2 cell differentiation take at least 7 days to appear after infection and 4–7 days after immunization *in vitro*, which provides sufficient time for the recruitment of basophils to the draining lymph node and the differentiation of B cells for the production of IgE in spleen. Experiments using proteases indicated that the contribution that basophils make to Th2 cell presentation of antigens to naïve T cells depends on allergen size^36^, although this is still controversial. Furthermore, Talay, O. *et al*. revealed that IgE levels peaked 15 days after the first infection with *N. brasiliensis* via the generation of IgE-switched B cells. At the point the infection was repeated (28 days later), the IgE levels had reached steady state levels. The second infection induced a second IgE peak 10 days later^24^. The fact that DNFB-induced IgE levels take ~5 days to peak may reflect the splenic response to hapten sensitization: it takes several days for B cells to start producing IgE. Indeed, in animals immunized with OVA, it takes at least 5 days for IgE production to become detectable and peak IgE levels are only detected ~10 days after the immunization^38,39^. These and our observations indicate that the most appropriate interval between hapten sensitizations is 14 days, as this gives the B cells enough time to become activated, produce IgE, and then become inactive. We are currently testing this notion with cell staining and quantitative PCR assays of the B cells in the spleen. Our approach might accelerate investigations into how and where IgE production is regulated as well as into ways to reduce stress and pain in mice.

The third DNFB sensitization in Protocol 5 yielded higher IgE levels than the second sensitization (Fig. 3). However, the third sensitization associated with almost the same expression of inflammatory cytokines as the second sensitization. In addition, the second sensitization induced AD-like skin phenotypes. Thus, the optimal period to evaluate the action of a candidate therapeutic drug against AD would be between the second and third sensitizations. Evaluation could involve observing histological features of AD-like skin lesions as well as measuring cytokine expression.

In conclusion, this study provides new perspectives in the development of drugs for AD. We hope that the lessons learned from this study will help other laboratories to adapt their drug development animal protocols so that the animals have the least possible pain and stress.

## Materials and Methods

### Animals

The animals were 6–7-week-old female BALB/c mice that were purchased from Clea Japan. The mice were housed under conventional conditions, namely, a constant temperature of 23 ± 2 °C and 60 ± 5% humidity with a regular 12 h light and dark cycle. Standard chow (CE-2, Clea Japan) and water were freely available. All experiments were conducted in accordance with the 2006 Japanese Association for Laboratory Animal Science guidelines for the care and use of experimental animals. The experimental protocols were also approved by the Animal Experiment Committee of Osaka University (Approval No. 25-1-1).

### Sensitization of mice with DNFB

After anesthetization, the dorsal skin of the BALB/c mice was shaved and tape stripped to disrupt the skin barrier. DNFB (Tokyo Chemical Industry) was then applied on a 1 × 1 cm square of the dorsal skin of each mouse according to one of five protocols. DNFB or vehicle patch was removed 2 h later. Protocol 1 is a conventional regimen where the dorsal skin was painted once weekly for 5 weeks (*i.e*., five painting sessions; Fig. 1a). Protocols 2, 3, and 4 involved painting the dorsal skin once weekly for 3 weeks, followed by a fourth painting session 9 days later (at day 23) and a fifth session 12 days (day 35, Protocol 2), 19 days (day 42, Protocol 3), or 26 days (day 49, Protocol 4) after the fourth session (*i.e*., Protocols 2–4 also involved five painting sessions; Fig. 2a). In Protocol 5, the dorsal skin was painted three times at intervals of 2 weeks.

### ELISA

About 100 µl of blood were collected from the legs using Goldenrod ANIMAL LANCET (Bio Research Center) and transferred into BD Microtainer Tubes (Becton, Dickinson and Company). The blood was allowed to stand for 15–30 min at room temperature before centrifugation at 7,900 g for 2 min. Sera were obtained and stored at −80 °C prior to analysis. The total IgE antibody levels in the sera were quantified by ELISA (BD Biosciences).

### Scratching Behavior

Scratching behavior of mice was recorded for 15 min. The record was performed 2 h after DNFB sensitization in the observation chamber using a digital video camera (CASIO EXILIM). Mice scratched themselves too many times for 1 s to count them. Therefore, multiple scratching behavior around the rostral shaved area using hind paws was counted as one event. The movie was played back at the slower speed using Kinovea.

### Histological analysis

In the Protocol 5-treated mice, dorsal skin tissue was removed before and 1–5 days after the second painting session. The excised skin specimens were fixed in 10% neutral formalin (Wako Pure Chemical Industries) or 4% paraformaldehyde (nacalai tesque) and embedded in paraffin (nacalai tesque). Sections (6 µm thick) were prepared and stained with hematoxylin and eosin (Wako Pure Chemical Industries) or toluidine blue (Sigma). Toluidine blue specifically stains mast cells. The definition of lymphocyte is described in the legend of Supplementary Fig. S4.

### Equipment and settings

The sections were observed under a brightfield microscope (OLYMPUS BX50; Olympus) equipped with a CCD camera (KEYENCE VB-7010; KEYENCE). The images were displayed using FLUORESCENCE DIGTAL MICROSCOPE CAMARA CONTROLLER VB-7000 (KEYENCE). All images were analyzed using ImageJ. Epidermal hypertrophy of H&E stained images was calculated by using a scale bar (×200, Supplementary Fig. S2). The numbers of infiltrated lymphocytes and mast cells were expressed as mean total counts in six high-power fields (HPFs; ×600, Supplementary Fig. S4) of 200,000 µm^2^ and one HPF (×200, Supplementary Fig. S3) of 400,000 µm^2^, respectively.

### RNA extraction and real-time quantitative PCR

In the Protocol 5-treated mice, dorsal skin tissue was removed before and 24 h after the second painting session. A skin biopsy was undertaken over time under anesthetization and ranged in size from 1 mm to 3 mm. Total RNA was purified using Sepasol-RNA I Super G (Nacalai Tesque) and Direct-zol RNA MiniPrep (Zymo Research) according to the manufacturers’ instructions. Subsequently, 50–100 ng of RNA was reverse-transcribed to first strand cDNA using Superscript III Reverse Transcriptase (Invitrogen). RT-qPCR was then performed by placing 5 μL of a 1:~20 dilution of the cDNA into a well that contained 15 μL of reaction mix: the reaction mix included 0.04 μL of 100 μM primer sets, 0.4 μL of Rox High reagent, and 10 μL of KAPA SYBR Fast qPCR Kit (Nippon Genetics). Reactions were run in at least duplicate on a StepOnePlus real-time PCR machine (Life Technologies) with the following protocol: 95 °C (30 s) followed by 40 cycles of 95 °C (5 s) and 60 °C (30 s). Data were normalized for the amount of Spt5 using the ΔΔCt method and a sample from the vehicle group was used as a calibrator. Primer sequences are listed on supplementary Table S4.

### Statistics

Statistical comparisons among groups were made by using R software (https://www.R-project.org/). Statistically significant differences were determined using one-way analysis of variance (ANOVA) with Tukey honestly significant difference (HSD) post hoc analysis. *P* values of less than 0.05 were considered significant and statistical significances at *P* < 0.05 and <0.01 are indicated by respective symbols in the figures.

All data generated or analyzed during this study are included in this published article and its Supplementary Information files.

## Electronic supplementary material

Supplementary Information 
