# Supplementary material for: A murine model of atopic dermatitis can be generated by painting the dorsal skin with hapten twice 14 days apart
Source: Sci Rep. 2018 Apr 16;8:5988. doi: 10.1038/s41598-018-24363-6 (PMC5902631; doi:10.1038/s41598-018-24363-6)
Supplement: Supplementary file 1 — Supplementary Information [file 41598_2018_24363_MOESM1_ESM.doc]

A murine model of atopic dermatitis can be generated by painting the dorsal skin with hapten twice 14 days apart

Ayaka Kitamura^1,2,3^, Ryohei Takata^1,2,3^, Shin Aizawa^4^, Hajime Watanabe^1,2^, and Tadashi Wada^1*^.

^1^Nucleic Acid Regulation (Yoshindo) Joint Research Laboratory, Department of Biotechnology, Osaka University, Osaka, Japan; ^2^Bioenvironmental Science, Department of Biotechnology, Osaka University, Osaka, Japan; ^3^Department of Research and Development, Yoshindo Inc., Toyama, Japan; ^4^Department of Functional Morphology, Nihon University School of Medicine, Tokyo, Japan.

*Corresponding author

*Correspondence to tadashi_wada@bio.eng.osaka-u.ac.jp

**Supplementary figures and table**

**Supplementary Figure. 1**

**
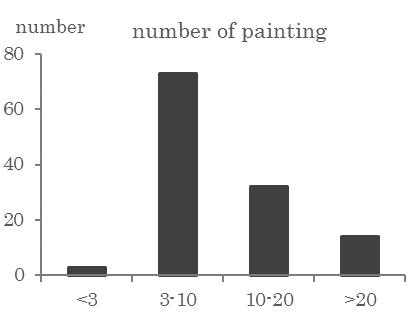
**

A

**
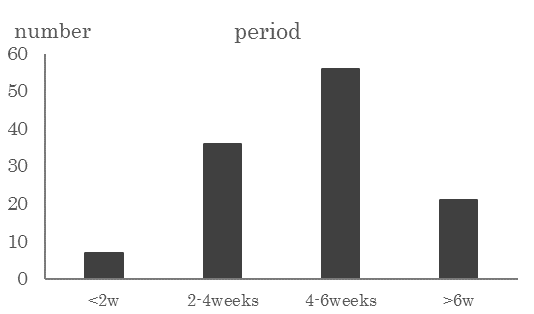
**

B

**Supplementary Figure 1.** Generation of a mouse model of atopic dermatitis using either DNFB or DNCB. The number of DNFB or DNCB paining and weeks (period) are indicated to generate the AD model mouse in (A) and (B), respectively. Vertical lines indicate the number of papers. These graphs were made based on data from papers as followed;

1. Bak, J.P. et al. Application of concentrated deep sea water inhibits the development of atopic dermatitis-like skin lesions in NC/Nga mice. BMC Complement. Altern. Med. 12, 108 (2012).

2. Bäumer, W. et al. TARC and RANTES, but not CTACK, are induced in two models of allergic contact dermatitis. Effects of cilomilast and diflorasone diacetate on T-cell-attracting chemokines. Br. J. Dermatol. 151, 823–830 (2004).

3. Beriat, G. K. et al. Is pimecrolimus cream (1%) an appropriate therapeutic agent for the treatment of external ear atopic dermatitis? Med. Sci. Monit. 18, BR135-43 (2012).

4. Bruno, A. et al. Fluoxetine Ameliorates Atopic Dermatitis-Like Skin Lesions in BALB/c Mice through Reducing Psychological Stress and Inflammatory Response. Z Front. Pharmacol 7, 1–9 (2016).

5. Caglayan Sozmen, S. et al. Resveratrol ameliorates 2,4-dinitrofluorobenzene-induced atopic dermatitis-like lesions through effects on the epithelium. PeerJ 4, e1889 (2016).

6. Chan, C. C. et al. Effect of dehydroepiandrosterone on atopic dermatitis-like skin lesions induced by 1-chloro-2,4-dinitrobenzene in mouse. J. Dermatol. Sci. 72, 149–157 (2013).

7. Cha, H. Y. et al. Hataedock Treatment Has Preventive Therapeutic Effects in Atopic Dermatitis-Induced NC/Nga Mice under High-Fat Diet Conditions. Evidence-based Complement. Altern. Med. 2016, (2016)

8. Cho, Y. S. et al. Identification of 4-[4-(4-fluoro-phenyl)-thiazol-2-ylamino]-2,6-dimethyl- phenol (KR-33749) as an inhibitor of 5-lipoxygenase with potent antiinflammatory activity. Pharmacology 86, 65–72 (2010).

9. Choi, E. J. et al. Heat-killed enterococcus faecalis EF-2001 ameliorates atopic dermatitis in a murine model. Nutrients 8, (2016).

10. Choi, E. J. et al. DA-9601 suppresses 2, 4-dinitrochlorobenzene and dust mite extract-induced atopic dermatitis-like skin lesions. Int. Immunopharmacol. 11, 1260–1264 (2011).

11. Choi, E. J. et al. Suppression of dust mite extract and 2,4-dinitrochlorobenzene-induced atopic dermatitis by the water extract of Lindera obtusiloba. J. Ethnopharmacol. 137, 802–807 (2011).

12. Choi, E. J. et al. Chemical composition and inhibitory effect of Lentinula edodes ethanolic extract on experimentally induced atopic dermatitis in vitro and in vivo. Molecules 21, (2016).

13. Choi, J. H. et al. Platycodon grandiflorum root-derived saponins attenuate atopic dermatitis-like skin lesions via suppression of NF-κB and STAT1 and activation of Nrf2/ARE-mediated heme oxygenase-1. Phytomedicine 21, 1053–1061 (2014).

14. Choi, J. H. et al. Cultivated ginseng inhibits 2,4-dinitrochlorobenzene-induced atopic dermatitis-like skin lesions in NC/Nga mice and TNF-alpha/IFN-gamma-induced TARC activation in HaCaT cells. Food Chem. Toxicol. 56, 193–203 (2013).

15. Choi, J. H. et al. Inhibitory effect of Psidium guajava water extract in the development of 2,4-dinitrochlorobenzene-induced atopic dermatitis in NC/Nga mice. Food Chem. Toxicol. 50, 2923–2929 (2012).

16. Choi, J. et al. Inhibition of inflammatory reactions in 2,4-Dinitrochlorobenzene induced Nc/Nga atopic dermatitis mice by non-thermal plasma. Sci. Rep. 6, 27376 (2016).

17. Choi, J. K. & Kim, S. H. Inhibitory effect of galangin on atopic dermatitis-like skin lesions. Food Chem. Toxicol. 68, 135–141 (2014).

18. Choi, J. K. et al. Oleanolic acid acetate inhibits atopic dermatitis and allergic contact dermatitis in a murine model. Toxicol. Appl. Pharmacol. 269, 72–80 (2013).

19. Choi, Y. Y. et al. Effect of dangguibohyul-tang, a mixed extract of astragalus membranaceus and angelica sinensis, on allergic and inflammatory skin reaction compared with single extracts of astragalus membranaceus or angelica sinensis. Evidence-based Complement. Altern. Med. 2016, (2016).

20. Choi, Y. Y. et al. Effect of dangguibohyul-tang, a mixed extract of astragalus membranaceus and angelica sinensis, on allergic and inflammatory skin reaction compared with single extracts of astragalus membranaceus or angelica sinensis. Evidence-based Complement. Altern. Med. 2016, (2016).

21. Choi, Y. Y. et al. Schizonepeta tenuifolia inhibits the development of atopic dermatitis in mice. Phyther. Res. 27, 1131–1135 (2013).

22. Choi, Y. Y. et al. Topical application of Kochia scoparia inhibits the development of contact dermatitis in mice. J. Ethnopharmacol. 154, 380–385 (2014).

23. Dang, L. et al. Role of the complement anaphylatoxin C5a-receptor pathway in atopic dermatitis in mice. Mol. Med. Rep. 11, 4183–4189 (2015).

24. Fang, L. W. et al. Danggui buxue tang inhibits 2,4-dinitrochlorobenzene: Induced atopic dermatitis in mice. Evidence-based Complement. Altern. Med. 2015, (2015).

25. Fujii, Y. et al. Repeated topical application of glucocorticoids augments irritant chemical-triggered scratching in mice. Arch. Dermatol. Res. 302, 645–652 (2010).

26. Fujiwara, R. et al. 2,4-Dinitrofluorobenzene-induced contact hypersensitivity response in NC/Nga mice fed fructo-oligosaccharide. J. Nutr. Sci. Vitaminol. (Tokyo). 56, 260–5 (2010).

27. Han, H. M. et al. Ameliorative effects of Artemisia argyi Folium extract on 2,4-dinitrochlorobenzene-induced atopic dermatitis-like lesions in BALB/c mice. Mol. Med. Rep. 14, 3206–3214 (2016).

28. Han, N. R. et al. Genuine traditional Korean medicine, Naju Jjok (Chung-Dae, Polygonum tinctorium) improves 2,4-dinitrofluorobenzene-induced atopic dermatitis-like lesional skin. Phytomedicine 21, 453–460 (2014).

29. Han, N. R. et al. Tryptanthrin ameliorates atopic dermatitis through down-regulation of TSLP. Arch. Biochem. Biophys. 542, 14–20 (2014).

30. Han, N. R. et al. Effect of Pyeongwee-San (KMP6) on 2,4-dinitrofluorobenzene-induced atopic dermatitis-like skin lesions in NC/Nga mice. Life Sci. 90, 147–153 (2012).

31. Han, S. C. et al. Fermented fish oil suppresses T helper 1/2 cell response in a mouse model of atopic dermatitis via generation of CD4+ CD25+ Foxp3+ T cells. BMC Immunol. 13, 1–12 (2012).

32. Heo, J. C. et al. Alleviation of atopic dermatitis-related symptoms by Perilla frutescens Britton. Int. J. Mol. Med. 28, 733–737 (2011).

33. Heo, J.C. et al. A Derivative of L-Allo Threonine Alleviates 2,4-Dinitrofluorobenzene-Induced Atopic Dermatitis Indications. Biosci. Biotechnol. Biochem. 76, 2021–2025 (2012).

34. Hong, S. H. et al. Quantitative determination of 12-hydroxyeicosatetraenoic acids by chiral liquid chromatography tandem mass spectrometry in a murine atopic dermatitis model. J. Vet. Sci. 16, 307–315 (2015).

35. Hussain, Z. et al. Efficient immuno-modulation of TH1/TH2 biomarkers in 2,4-dinitrofluorobenzene-induced atopic dermatitis: Nanocarrier-mediated transcutaneous co-delivery of anti-inflammatory and antioxidant drugs. PLoS One 9, (2014).

36. Hussain, Z. et al. Downregulation of immunological mediators in 2,4-dinitrofluorobenzene-induced atopic dermatitis-like skin lesions by hydrocortisoneloaded chitosan nanoparticles. Int. J. Nanomedicine 9, 5143–5156 (2014).

37. Hwang, J. S. et al. Topical application of Taglisodog-eum inhibits the development of experimental atopic dermatitis. J Ethnopharmacol 145, 536–546 (2013).

38. Hwang, J. S. et al. Modulation of experimental atopic dermatitis by topical application of Gami-Cheongyeul-Sodok-Eum. 1–10 (2013).

39. Hwang, J. et al. Immunomodulatory effect of water soluble extract separated from mycelium of Phellinus linteus on experimental atopic dermatitis. BMC Complement. Altern. Med. 12, 159 (2012).

40. Ikeda, Y. et al. Administration of substance P during a primary immune response amplifies the secondary immune response via a long-lasting effect on CD8+ T lymphocytes. Arch. Dermatol. Res. 299, 345–351 (2007).

41. Im, L. R. et al. Inhibitory effect of Kyungohkgo in the development of 2,4- dinitrochlorobenzene-induced atopic dermatitis in NC/Nga mice. Arch. Pharm. Res. 34, 317–321 (2011).

42. Inagaki, N. et al. Inhibition of scratching behavior associated with allergic dermatitis in mice by tacrolimus, but not by dexamethasone. Eur. J. Pharmacol. 546, 189–196 (2006).

43. Inagaki, N. et al. Depletion of substance P, a mechanism for inhibition of mouse scratching behavior by tacrolimus. Eur. J. Pharmacol. 626, 283–289 (2010).

44. Jang, A. H. et al. Rosmarinic acid attenuates 2,4-dinitrofluorobenzene-induced atopic dermatitis in NC/Nga mice. Int. Immunopharmacol. 11, 1271–1277 (2011).

45. Jeon, Y.-D. et al. Effects of Ixeris dentata water extract and caffeic acid on allergic inflammation in vivo and in vitro. BMC Complement. Altern. Med. 15, 196 (2015).

46. Jung, B. G. et al. Fermented Maesil (Prunus mume) with probiotics inhibits development of atopic dermatitis-like skin lesions in NC/Nga mice. Vet. Dermatol. 21, 184–191 (2010).

47. Jung, J. W. et al. Ribes fasciculatum var. chinense attenuated allergic inflammation in vivo and in vitro. Biomol. Ther. 22, 547–552 (2014).

48. Jung, K. H. et al. Bee Venom Phospholipase A2 Ameliorates House Dust Mite Extract Induced Atopic Dermatitis Like Skin Lesions in Mice. Toxins (Basel). 9, 68 (2017).

49. Kang, G. J. et al. The inhibitory effect of premature citrus unshiu extract on atopic dermatitis in vitro and in vivo. Toxicol. Res. 27, 173–180 (2011).

50. Kang, M. & Choung, S. Y. Solanum tuberosum L. cv Hongyoung extract inhibits 2,4-dinitrochlorobenzene-induced atopic dermatitis in NC/Nga mice. Mol. Med. Rep. 14, 3093–3103 (2016).

51. Karki, R. et al. Inhibitory effect of Nelumbo nucifera (Gaertn.) on the development of atopic dermatitis-like skin lesions in NC/Nga mice. Evidence-based Complement. Altern. Med. 2012, (2012).

52. Ki, N. Y. et al. The Hot-Water Extract of Smilacis Chinae Rhizome Suppresses 2,4-Dinitrochlorobenzene and House Dust Mite-Induced Atopic Dermatitis-Like Skin Lesions in Mice. Phyther. Res. 30, 636–645 (2016).

53. Kim, C. G. et al. Bathing Effects of Various Seawaters on Allergic (Atopic) Dermatitis-Like Skin Lesions Induced by 2,4-Dinitrochlorobenzene in Hairless Mice. Evidence-based Complement. Altern. Med. 2015, (2015).

54. Kim, D. Y. et al. Oral administration of Uncariae rhynchophylla inhibits the development of DNFB-induced atopic dermatitis-like skin lesions via IFN-gamma down-regulation in NC/Nga mice. J. Ethnopharmacol. 122, 567–572 (2009).

55. Kim, D. et al. Differential expression of cell surface markers in response to 2,4-dinitrofluorobenzene in RAW 264.7 and primary immune cells. BMB Rep. 45, 538–543 (2012).

56. Kim, G. D. et al. Immune response against 2,4-dinitrofluorobenzene-induced atopic dermatitis-like clinical manifestation is suppressed by spermidine in NC/Nga mice. Scand. J. Immunol. 81, 221–228 (2015).

57. Kim, G. D. et al. α-Lipoic acid suppresses the development of DNFB-induced atopic dermatitis-like symptoms in NC/Nga mice. Exp. Dermatol. 20, 97–101 (2011).

58. Kim, G. D. et al. Immunosuppressive effects of fisetin against dinitrofluorobenzene-induced atopic dermatitis-like symptoms in NC/Nga mice. Food Chem. Toxicol. 66, 341–349 (2014).

59. Kim, G. D. et al. Aspartame Attenuates 2, 4-Dinitrofluorobenzene-Induced Atopic Dermatitis–Like Clinical Symptoms in NC/Nga Mice. J. Invest. Dermatol. 135, 2705–2713 (2015).

60. Kim, H. et al. Effects of Oral Intake of Kimchi -Derived Lactobacillus plantarum K8 Lysates on Skin Moisturizing. 25, 74–80 (2015).

61. Kim, H. et al. 7,8,4'-Trihydroxyisoflavone attenuates DNCB-induced atopic dermatitis-like symptoms in NC/Nga mice. PLoS One 9, (2014).

62. Kim, H. R. et al. Hyperoxygenation attenuated a murine model of atopic dermatitis through raising skin level of ROS. PLoS One 9, (2014).

63. Kim, I. S. et al. (S)-(+)-decursin derivative, (S)-(+)-3-(3,4-dihydroxy-phenyl)-acrylic acid 2,2-dimethyl-8-oxo-3,4-dihydro-2H,8H-pyrano[3,2-g]-chromen-3-yl-ester, attenuates the development of atopic dermatitis-like lesions in NC/Nga mice. Mol. Biol. Rep. 40, 2541–2548 (2013).

64. Kim, I. S. et al. Inhibitory effect of arazyme on the development of atopic dermatitis-like lesions in BALB/c and Nc/Nga mice. Mol. Med. Rep. 11, 3995–4001 (2015).

65. Kim, I. S. et al. Effect of (E)-2-(3,4-dimethoxyphenyl)-4-oxo-4H-chromen-7-yl-3-(3,4- dimethoxyphenyl) acrylate on the development of atopic dermatitis-like lesions. Life Sci. 91, 338–344 (2012).

66. Kim, J. H. et al. Effects of topical application of Astragalus membranaceus on allergic dermatitis. Immunopharmacol. Immunotoxicol. 35, 151–6 (2013).

67. Kim, M. J. & Choung, S. Y. Mixture of polyphenols and anthocyanins from vaccinium uliginosum L. Alleviates DNCB-induced atopic dermatitis in NC/Nga mice. Evidence-based Complement. Altern. Med. 2012, (2012).

68. Kim, M. S. et al. A probiotic preparation alleviates atopic dermatitis-like skin lesions in murine models. Toxicol. Res. 32, 149–158 (2016).

69. Kim, S. H. et al. High-intensity swimming exercise increases dust mite extract and 1-chloro-2,4-dinitrobenzene-derived atopic dermatitis in BALB/c mice. Inflammation 37, 1179–1185 (2014).

70. Kim, S. R. et al. Topical application of herbal mixture extract inhibits ovalbumin- or 2,4-dinitrochlorobenzene-induced atopic dermatitis. Evidence-based Complement. Altern. Med. 2012, (2012).

71. Kim, S. R. et al. Oral administration of herbal mixture extract inhibits 2,4-dinitrochlorobenzene-induced atopic dermatitis in BALB/c mice. Mediators Inflamm. 2014, (2014).

72. Kim, S. Y. et al. Transduced PEP-1-FK506BP ameliorates atopic dermatitis in NC/Nga mice. J. Invest. Dermatol. 131, 1477–85 (2011).

73. Kim, T. H. et al. Melatonin inhibits the development of 2,4-dinitrofluorobenzene-induced atopic dermatitis-like skin lesions in NC/Nga mice. J. Pineal Res. 47, 324–329 (2009).

74. Kim, T. H. et al. The histone deacetylase inhibitor, trichostatin A, inhibits the development of 2,4-dinitrofluorobenzene-induced dermatitis in NC/Nga mice. Int. Immunopharmacol. 10, 1310–1315 (2010).

75. Kim, T. H. et al. The inhibitory effect of naringenin on atopic dermatitis induced by DNFB in NC/Nga mice. Life Sci. 93, 516–524 (2013).

76. Kim, T. H. et al. Omega-3 fatty acid-derived mediator, Resolvin E1, ameliorates 2,4-dinitrofluorobenzene-induced atopic dermatitis in NC/Nga mice. Int. Immunopharmacol. 14, 384–391 (2012).

77. Kim, W.-J. et al. Effects of Cymbidium Root Ethanol Extract on Atopic Dermatitis. Evidence-Based Complement. Altern. Med. 2016, 1–10 (2016).

78. Kim, W. Y. et al. A herbal formula, atofreellage, ameliorates atopic dermatitis-like skin lesions in an NC/Nga mouse model. Molecules 21, 1–13 (2016).

79. Kim, Y. et al. Celastrol binds to ERK and inhibits Fc epsilon RI signaling to exert an anti-allergic effect. Eur. J. Pharmacol. 612, 131–142 (2009).

80. Ku, J. M. et al. Effects of Angelicae dahuricae Radix on 2, 4-Dinitrochlorobenzene-Induced Atopic Dermatitis-Like Skin Lesions in mice model. BMC Complement. Altern. Med. 17, 98 (2017).

81. Lee, D. I. et al. Alternative therapeutic advantages of catfish bile on atopic dermatitis: Protection of T cell-mediated skin disease via antioxidant activities. J. Pharm. Pharmacol. 63, 1327–1335 (2011).

82. Lee, H. S. et al. Oral administration of 4-hydroxy-3-methoxycinnamaldehyde attenuates atopic dermatitis by inhibiting T cell and keratinocyte activation. PLoS One 10, 1–16 (2015).

83. Lee, J. H. et al. Capsiate Inhibits DNFB-Induced Atopic Dermatitis in NC/Nga Mice through Mast Cell and CD4+ T-Cell Inactivation. J. Invest. Dermatol. 135, 1977–85 (2015).

84. Lee, J. H. et al. Topical Application of Eupatilin Ameliorates Atopic Dermatitis-Like Skin Lesions in NC / Nga Mice. 29, 61–68 (2017).

85. Lee, J. S. et al. The inhibitory effect of duchesnea chrysantha extract on the development of atopic dermatitis-like lesions by regulating IgE and cytokine production in Nc/Nga mice. Phyther. Res. 26, 284–290 (2012).

86. Lee, K. H. et al. Hizikia fusiformis fractions successfully improve atopic dermatitis indices in anti-CD3-stimulated splenocytes and 2,4-dinitrochlorobenzene-treated BALB/c mice. J. Pharm. Pharmacol. 66, 466–476 (2014).

87. Lee, S. H. et al. Effect of German chamomile oil application on alleviating atopic dermatitis-like immune alterations in mice. J. Vet. Sci. 11, 35–41 (2010).

88. Lee, S-J. et al. Oral Administration of Astragalus membranaceus Inhibits the Development of DNFB-Induced Dermatitis in NC / Nga Mice. 30, 1468–1471 (2007).

89. Li, C. X. et al. Andrographolide suppresses thymic stromal lymphopoietin in phorbol myristate acetate/calcium ionophore A23187-activated mast cells and 2,4-dinitrofluorobenzene-induced atopic dermatitis-like mice model. Drug Des. Devel. Ther. 10, 781–791 (2016).

90. Li, Y. Z. et al. Anti-inflammatory effect of qingpeng ointment in atopic dermatitis-like murine model. Evidence-based Complement. Altern. Med. 2013, 1–8 (2013).

91. Lim, S. K. et al. Weissella cibaria WIKIM28 ameliorates atopic dermatitis-like skin lesions by inducing tolerogenic dendritic cells and regulatory T cells in BALB/c mice. Sci. Rep. 7, 40040 (2017).

92. Lim, S. J. et al. Effects of Hovenia dulcis Thunb. extract and methyl vanillate on atopic dermatitis-like skin lesions and TNF-alpha/IFN-gamma-induced chemokines production in HaCaT cells. J. Pharm. Pharmacol. 68, 1465–1479 (2016).

93. Lin, G. et al. 1 beta-Hydroxyalantolactone, a sesquiterpene lactone from Inula japonica, attenuates atopic dermatitis-like skin lesions induced by 2,4-dinitrochlorobenzene in the mouse. Pharm. Biol. 54, (2016).

94. Moon, J. S. et al. Topical application of a phospholipid mixture purified from pig lungs ameliorates 2,4-dinitrofluorobenzene-induced allergic contact dermatitis in BALB/c mice. Mol. Biol. Rep. 39, 4237–4247 (2012).

95. Nam, D. Y. et al. Mitigation of 2,4-dinitrofluorobenzene-induced atopic dermatitis-related symptoms by Terminalia chebula Retzius. Int. J. Mol. Med. 28, 1013–101

96. Newell, L. et al. Sensitization via healthy skin programs Th2 responses in individuals with atopic dermatitis. J. Invest. Dermatol. 133, 2372–80 (2013).

97. Nishimura, K. et al. Influence of Hot Spring Water on Fatty Acid Composition of Skin Surface Lipids in Hairless Mouse Model of Atopic Dermatitis. 39, 1718–1722 (2016).

98. Oh, S. R. et al. Betula platyphylla attenuated mast cell-mediated allergic inflammation in vivo and in vitro. Life Sci. 91, 20–28 (2012).

99. Park, H. S. et al. Functional polysaccharides from Grifola frondosa aqueous extract inhibit atopic dermatitis-like skin lesions in NC/Nga mice. Biosci. Biotechnol. Biochem. 79, 147–154 (2015).

100. Park, J. Y. et al. Effects of acupuncture on 1-chloro-2,4-dinitrochlorobenzene-induced atopic dermatitis. Evidence-based Complement. Altern. Med. 2013, 1–9 (2013).

101. Park, S. J. et al. Effect of nodakenin on atopic dermatitis-like skin lesions. Biosci. Biotechnol. Biochem. 78, 1568–1571 (2014).

102. Park, S. J. et al. Effect of eriodictyol on the development of atopic dermatitis-like lesions in ICR mice. Biol. Pharm. Bull. 36, 1375–9 (2013).

103. Park, S. J. et al. Topical application of Chrysanthemum indicum L. Attenuates the development of atopic dermatitis-like skin lesions by suppressing serum IgE levels, IFN-gamma , and IL-4 in Nc/Nga mice. Evidence-based Complement. Altern. Med. 2012, (2012).

104. Pokharel, Y. R. et al. Sopungyangjae-Tang inhibits development of dermatitis in Nc/Nga mice. Evidence-based Complement. Altern. Med. 5, 173–180 (2008).

105. Qi, X. F. et al. Effects of Bambusae caulis in Liquamen on the development of atopic dermatitis-like skin lesions in hairless mice. J. Ethnopharmacol. 123, 195–200 (2009).

106. Qiao, M. et al. Erratum to: The Natural Course of Atopic Dermatitis and the Association with Asthma. Inflammation 40, 546–554 (2017).

107. Roßbach, K. et al. Histamine H4 receptor antagonism reduces hapten-induced scratching behaviour but not inflammation. Exp. Dermatol. 18, 57–63 (2009). .

108. Saba, E. et al. Fermented rice bran prevents atopic dermatitis in DNCB-treated NC/Nga mice. J. Biomed. Res. 30, 334–343 (2016).

109. Samukawa, K. et al. Red Ginseng Inhibits Scratching Behavior Associated With Atopic Dermatitis in Experimental Animal Models. J. Pharmacol. Sci. 118, 391–400 (2012).

110. Schuepbach-Mallepell, S. et al. Antagonistic effect of the inflammasome on thymic stromal lymphopoietin expression in the skin. J. Allergy Clin. Immunol. 132, 1348–1357 (2013).

111. Shim, E. H. & Choung, S. Y. Inhibitory effects of S olanum tuberosum L. var. vitelotte extract on 2,4-dinitrochlorobenzene-induced atopic dermatitis in mice. J. Pharm. Pharmacol. 66, 1303–1316 (2014).

112. Shin, Y. K. et al. Cinnamomum cassia bark produced by solid-state fermentation with Phellinus baumii has the potential to alleviate atopic dermatitis-related symptoms. Int. J. Mol. Med. 35, 187–194 (2015)

113. Sohn, E. H. et al. Effects of Korean red ginseng extract for the treatment of atopic dermatitis-like skin lesions in mice. J. Ginseng Res. 35, 479–486 (2011).

114. Sun, G. et al. Tumor necrosis factor-alpha, monocyte chemoattractant protein-1 and intercellular adhesion molecule-1 increase during the development of a 2,4-dinitrofluorobenzene-induced immediate-type dermatitis in rats. Inflamm. Res. 62, 589–597 (2013).

115. Sun, M. et al. Suppression of 2,4-dinitrochlorobenzene-induced atopic dermatitis by extract of Bacillus Calmette-Guerin. Mol. Med. Rep. 9, 689–694 (2014).

116. Terakawa, M. et al. Oral chymase inhibitor SUN13834 ameliorates skin inflammation as well as pruritus in mouse model for atopic dermatitis. Eur. J. Pharmacol. 601, 186–191 (2008).

117. Tomimori, Y. et al. Repeated topical challenge with chemical antigen elicits sustained dermatitis in NC/Nga mice in specific-pathogen-free condition. J. Invest. Dermatol. 124, 119–124 (2005).

118. Ulker, O. C. et al. Evaluation of auricular lymph node cell lymphocyte proliferation and cytokine production as non-radioactive endpoints during murine contact allergy. J. Immunotoxicol. 8, 131–139 (2011).

119. Wang, L. H. et al. Curative effect of BCG-polysaccharide nuceic acid on atopic dermatitis in mice. Asian Pac. J. Trop. Med. 7, 913–917 (2014).

120. Wu, G. et al. Inhibition of 2,4-dinitrofluorobenzene-induced atopic dermatitis by topical application of the butanol extract of Cordyceps bassiana in NC/Nga mice. J. Ethnopharmacol. 134, 504–509 (2011).

121. Yamashita, H. et al. Comparison of the efficacy of tacrolimus and cyclosporine A in a murine model of dinitrofluorobenzene-induced atopic dermatitis. Eur. J. Pharmacol. 645, 171–176 (2010).

122. Yang, G. et al. Effect of Chrysanthemi borealis flos on atopic dermatitis induced by 1-chloro 2,4-dinitrobenzene in NC/Nga mouse. Immunopharmacol. Immunotoxicol. 34, 413–418 (2012).

123. Yang, G. et al. Inhibitory effects of Chelidonium majus extract on atopic dermatitis-like skin lesions in NC/Nga mice. J. Ethnopharmacol. 138, 398–403 (2011).

124. Yang, H. et al. Estimation of the environmental effect of natural volatile organic compounds from Chamaecyparis obtusa and their effect on atopic dermatitis-like skin lesions in mice. Mol. Med. Rep. 345–350 (2015). doi:10.3892/mmr.2015.3431

125. Yang, H. et al. Elemol from Chamaecyparis obtusa ameliorates 2,4-dinitrochlorobenzene-induced atopic dermatitis. Int. J. Mol. Med. 36, 463–472 (2015).

126. Yang, I. J. et al. Inhibitory Effect of Valencene on the Development of Atopic Dermatitis-Like Skin Lesions in NC/Nga Mice. Evid. Based. Complement. Alternat. Med. 2016, 9370893 (2016).

127. Yang, J. H. et al. Ethanol Extract of Sanguisorbae Radix Inhibits Mast Cell Degranulation and Suppresses 2,4-Dinitrochlorobenzene-Induced Atopic Dermatitis-Like Skin Lesions. Mediators Inflamm. 2016, (2016).

128. Yoon, H.J. et al. Protective effect of diet supplemented with rice prolamin extract against DNCB-induced atopic dermatitis in BALB/c mice. BMC Complement. Altern. Med. 15, 353 (2015).

129. Yoon, Y.S. et al. Positive Effects of Hydrogen Water on 2,4-Dinitrochlorobenzene-Induced Atopic Dermatitis in NC/Nga Mice. Biol. Pharm. Bull. 37, 1480–5 (2014).

130. Yuan, X. Y. et al. Topical application of aloperine improves 2,4-dinitrofluorobenzene-induced atopic dermatitis-like skin lesions in NC/Nga mice. Eur. J. Pharmacol. 658, 263–269 (2011).

131. Zhou, S. et al. Original article Sanpao herbs inhibit development of atopic dermatitis in Balb / c mice. 140–144

**Supplementary Figure 2.**


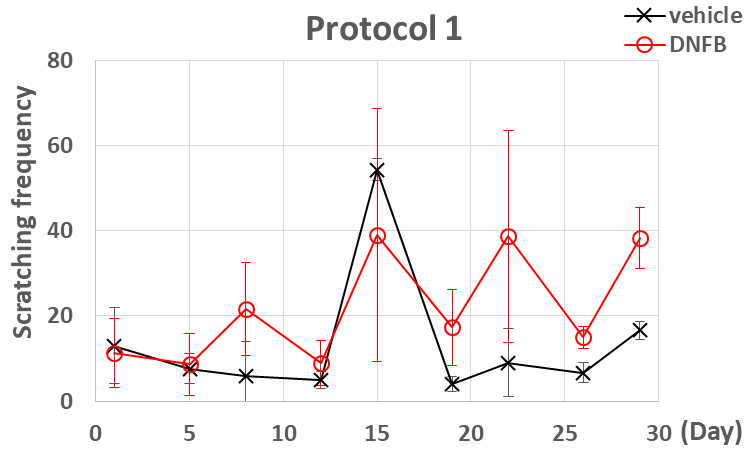
A


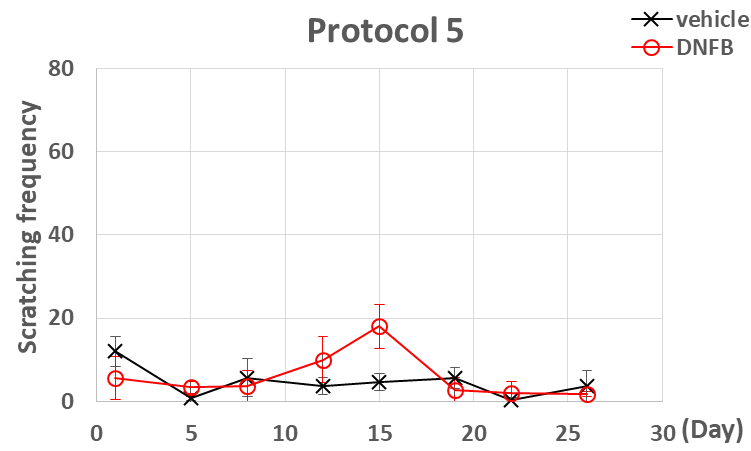
B

**Supplementary Figure 2.** The scratching frequency was counted twice a week and compared the duration of persistence of itching. Mice were observed for 15 min 1 day and 5 days after each DNFB application (A) and 1 day, 5 days, 8 days and 12 days in case of (B). Mean and s.d. is indicated (n=3). Arrows indicate the day of DNFB application. Scratching frequency was not increased after the first DNFB application in both protocols (day 1 and day 5). The second DNFB application induced increased scratching frequency in both models (A, day 8 and B, day 15). The absolute number of scratching behavior was similar in both models. After that, increased scratching behavior was observed 1 day after DNFB application in Protocol 1 (A, day 15, day 22 and day 29), although it demonstrated much individual differences. Mice showed less scratching behavior at 5 days after DNFB application (A, day 19 and day 26) than that at 1 day after DNFB application (A, day 15 and day 22). In addition, scratching behavior might increase even when vehicle was applied to mice especially at day 15 and day 29 (A).

**Supplementary Table 1.** The epidermal thickness of mice was measured using ImageJ. AD; identification of experiments, No; (identification of mouse)_(identification of slide), N; the number of fields, n; the number of mice.

**Supplementary Figure 3.**


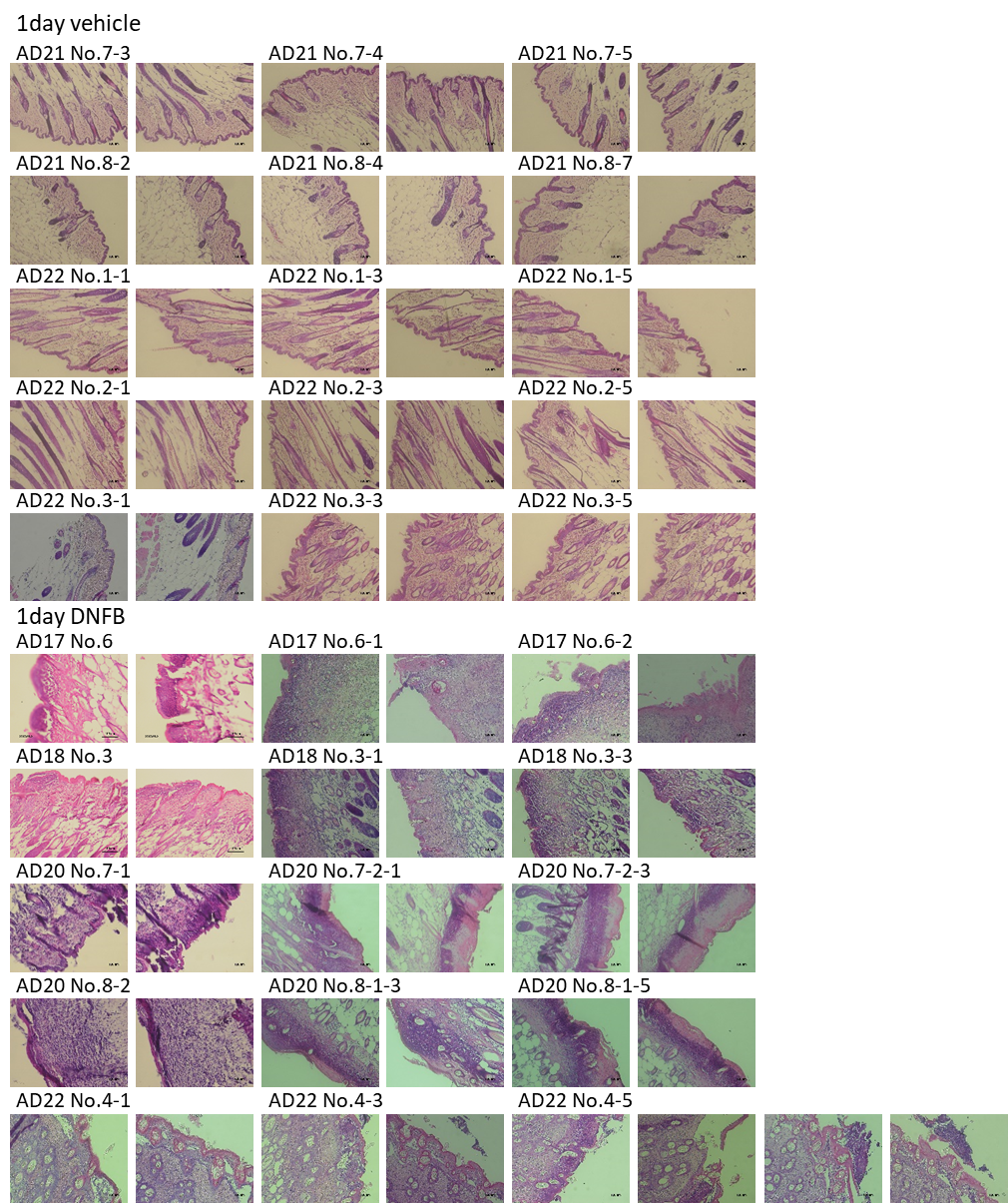


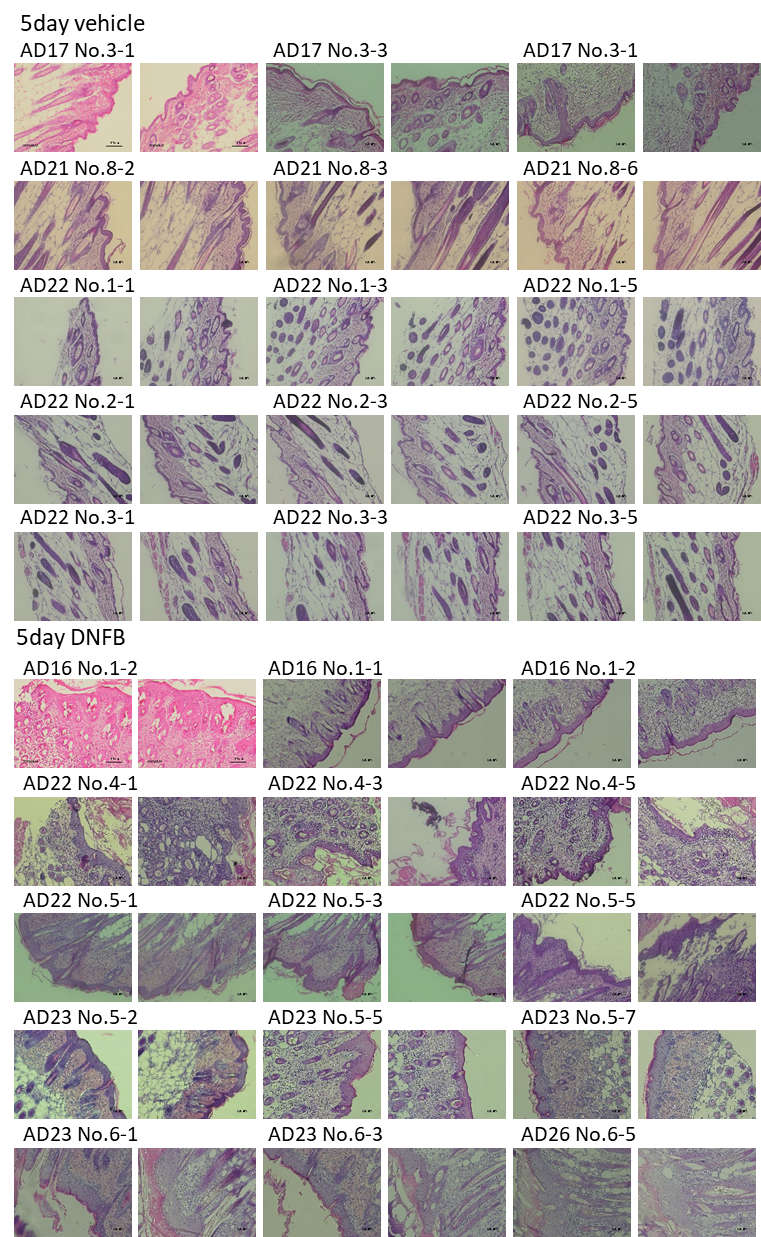


**Supplementary Figure 3.** Epidermal thickness of mice was measured using ImageJ. Mice were painted with vehicle or DNFB twice 14 days apart. The tissue was recovered 1 day or 5 day after 2^nd^ painting. Five mice were used for each data (n=5). Three different areas per field (×200) were measured. At least two fields were used per slide. Three slides were observed per mouse. The length (µm) was calculated using ImageJ.

**Supplementary Table 2.** The number of mast cells was counted. AD; identification of experiments, No; (identification of mouse)_(identification of slide), N; the number of fields, n; the number of mice.

**Supplementary Figure 4.**


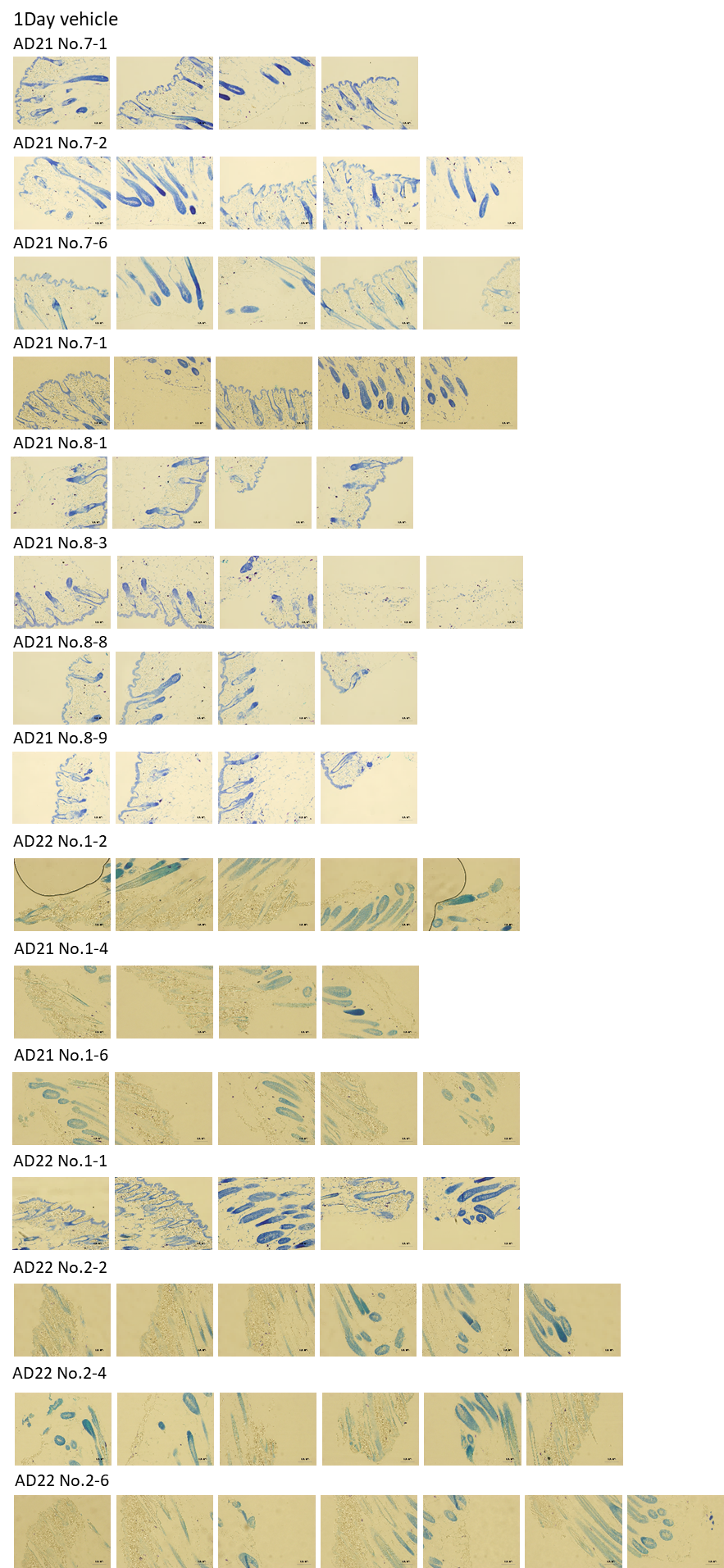


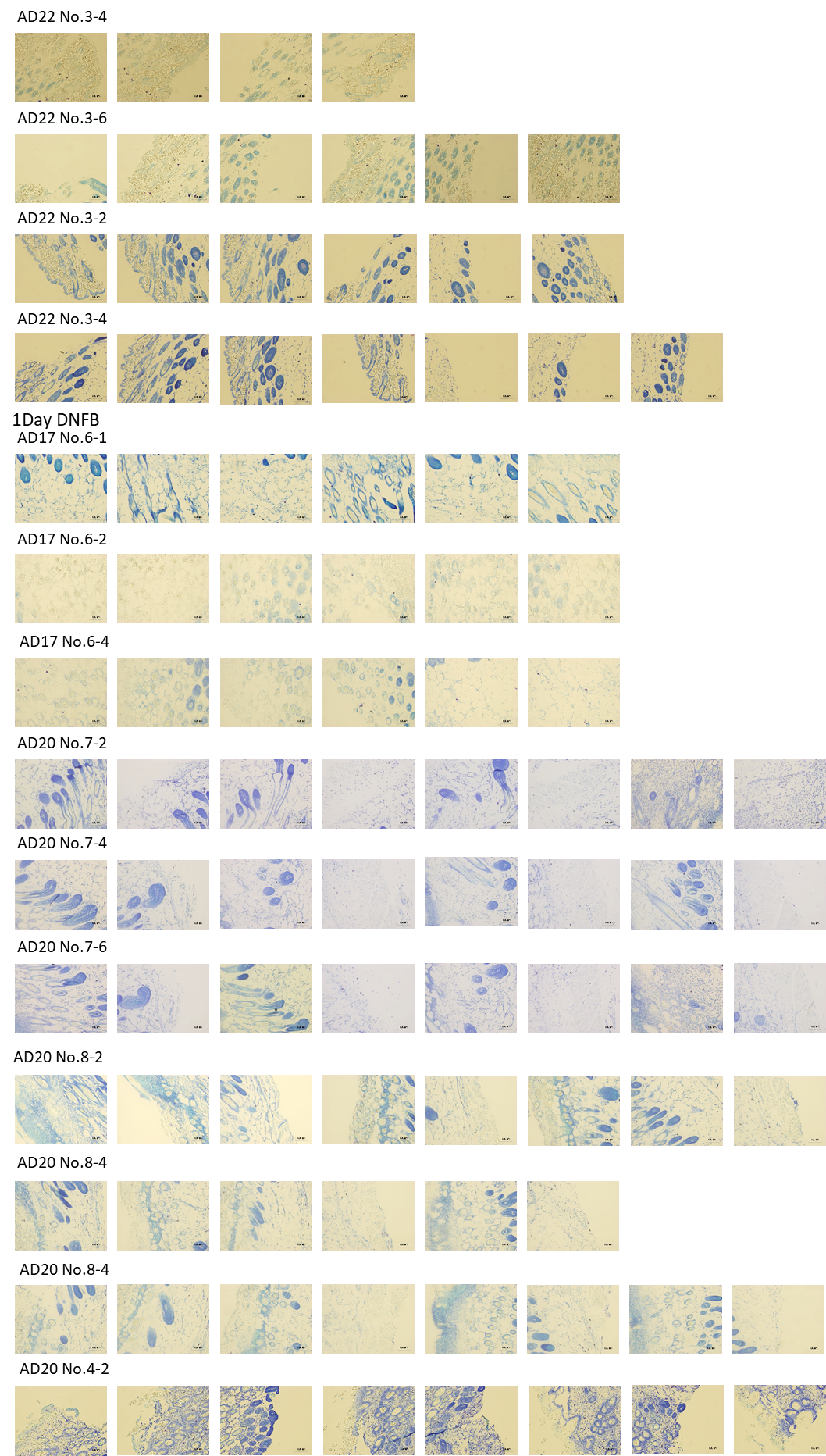


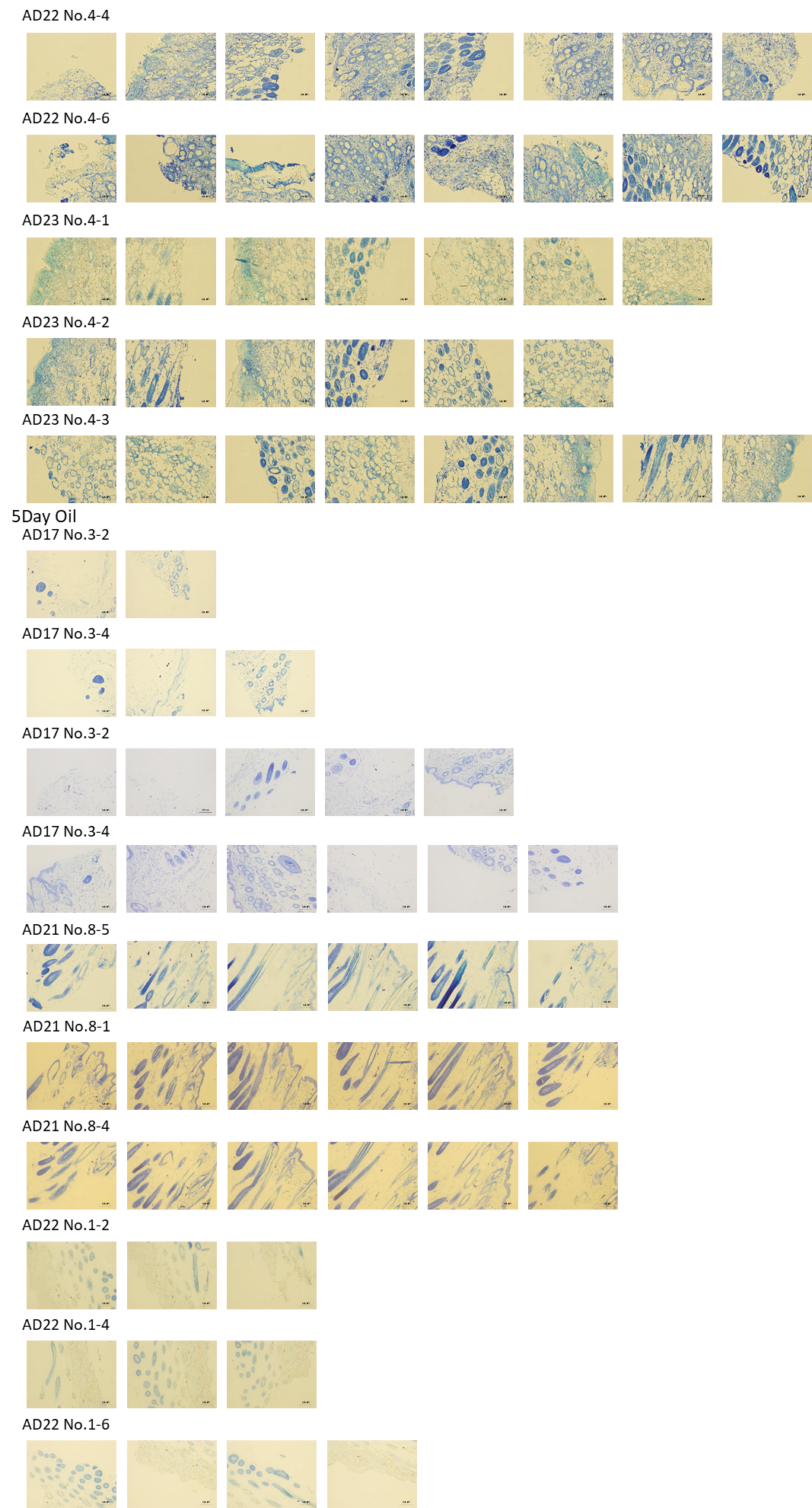


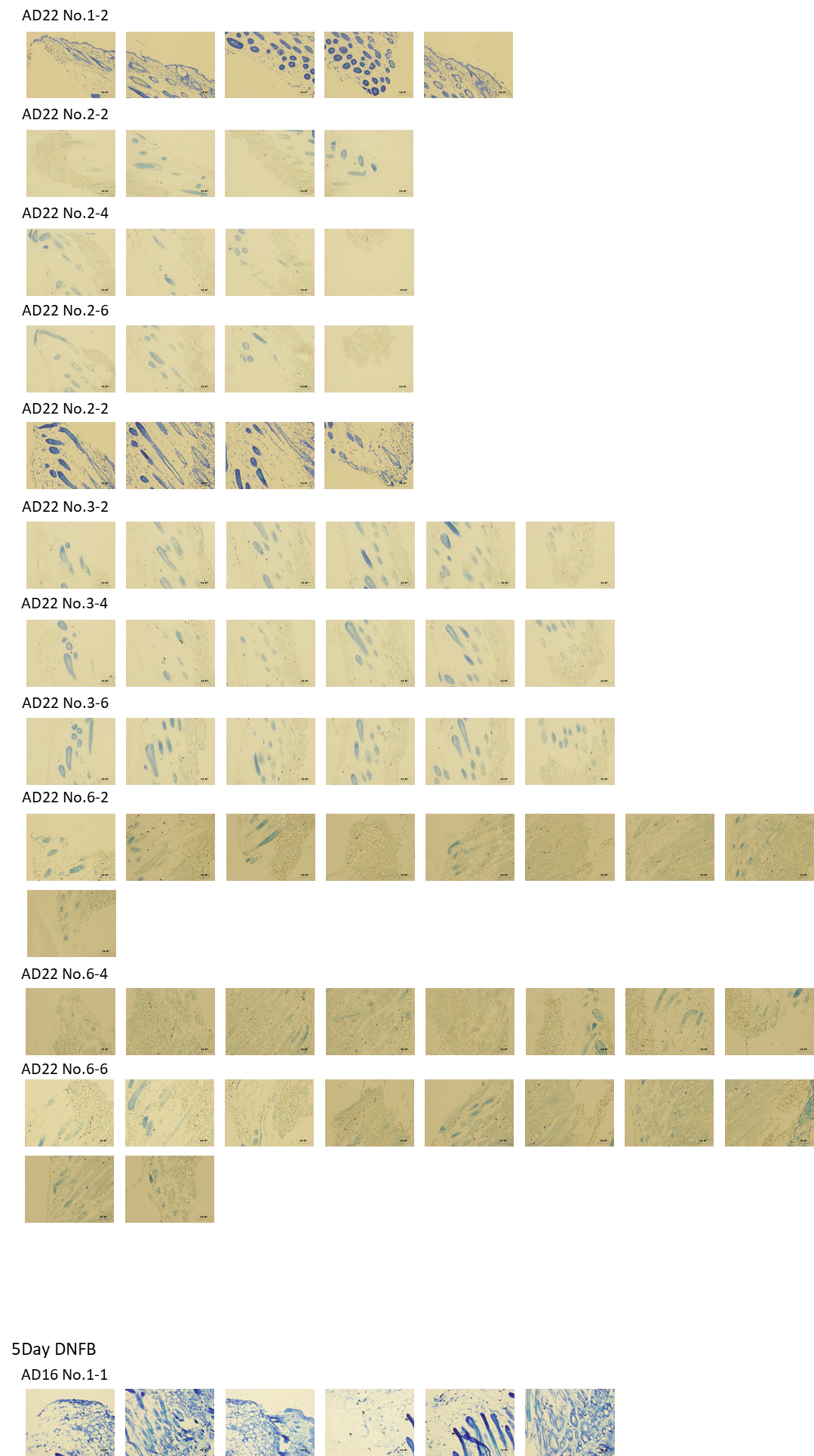


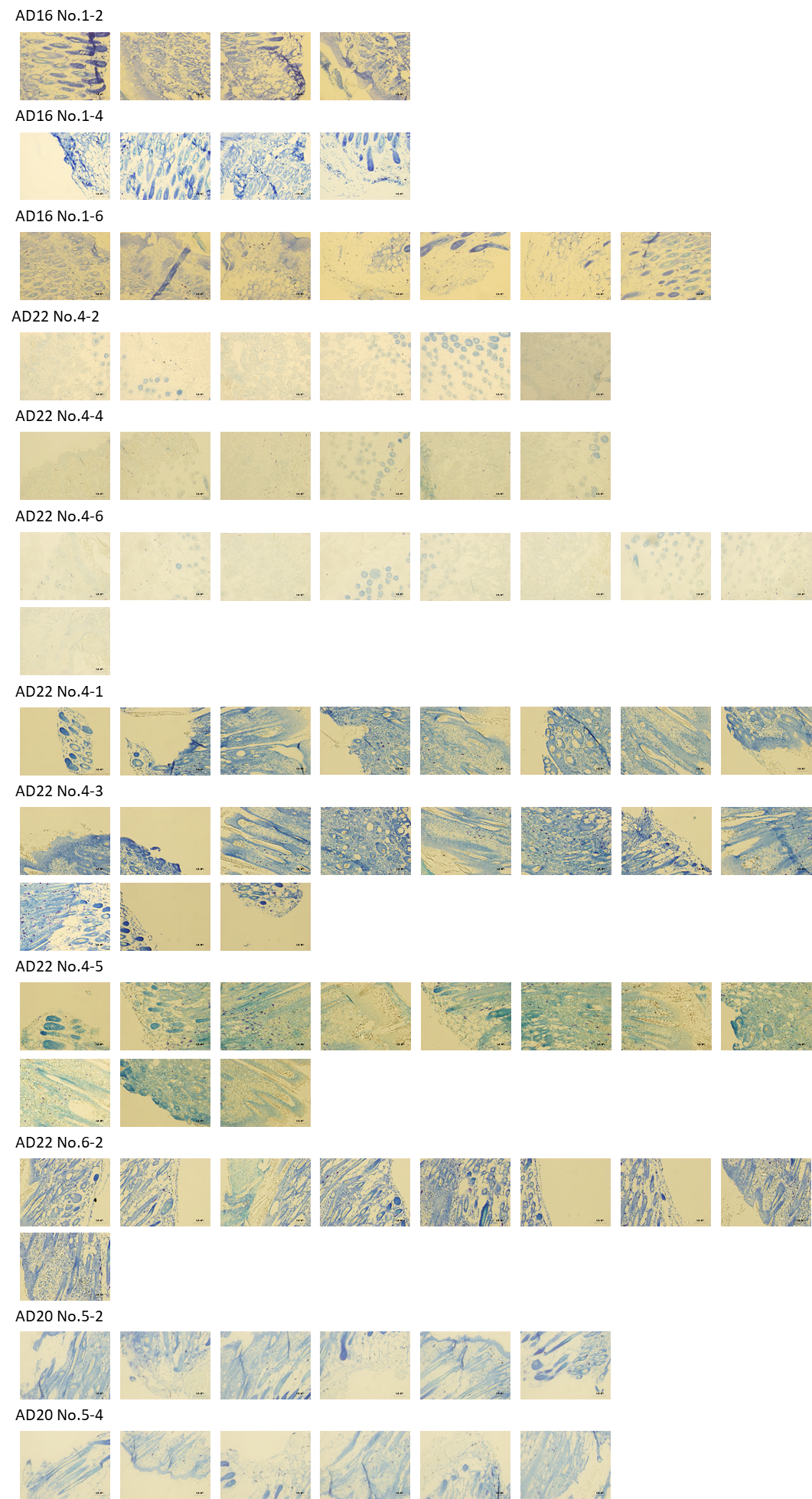


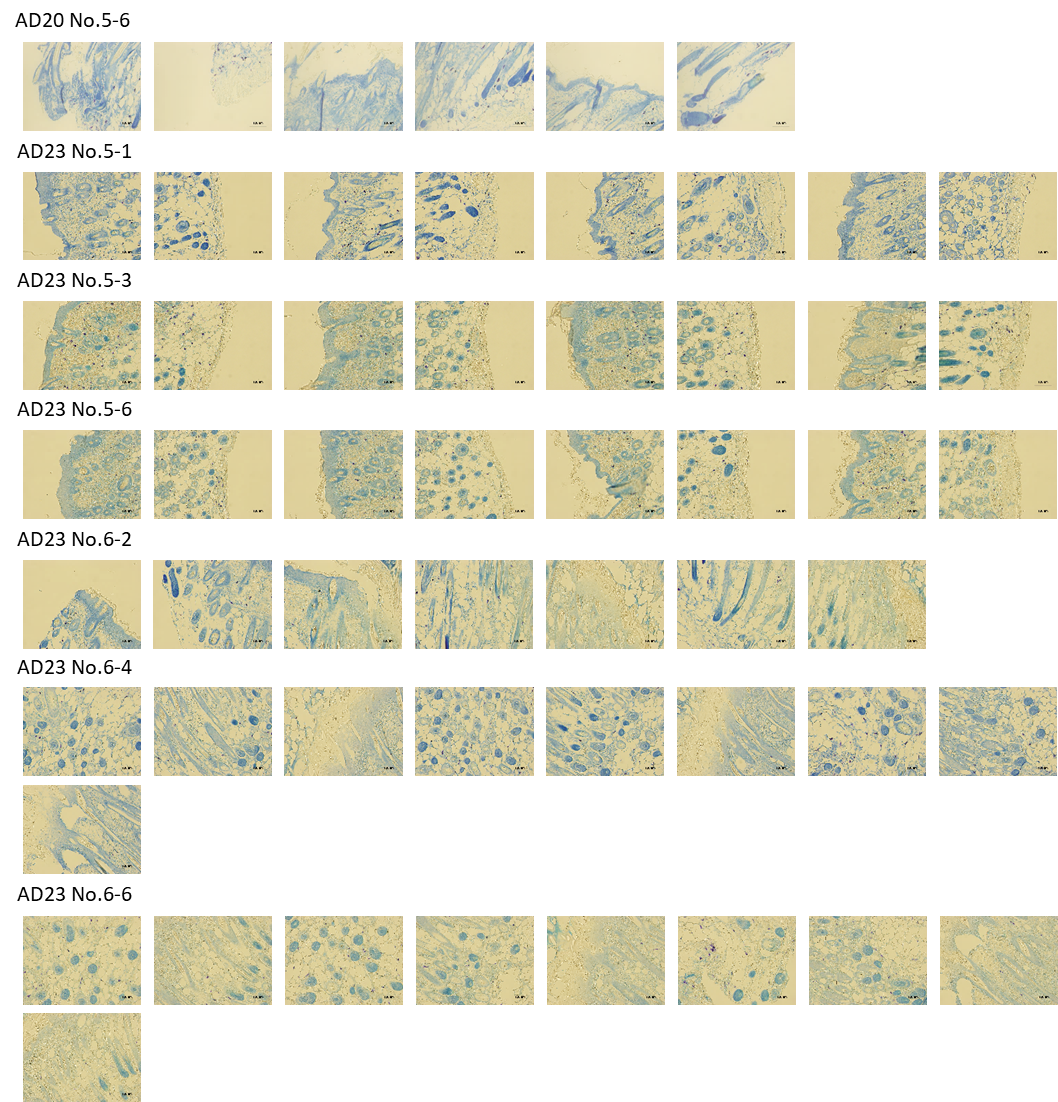


**Supplementary Figure 4.** The number of mast cells in dermis and subcutaneous tissues was counted. Mice were painted with vehicle or DNFB twice 14 days apart. The tissue was recovered 1 day or 5 day after 2^nd^ painting. Four to five mice were used for each data (n=4–5). At least three slides per mouse were used. Area (µm^2^) was calculated using ImageJ.

**Supplementary Table 3.** The number of lymphocytes in lesional skin was counted. AD; identification of experiments, No; (identification of mouse)_(identification of slide), N; the number of fields, n; the number of mice.


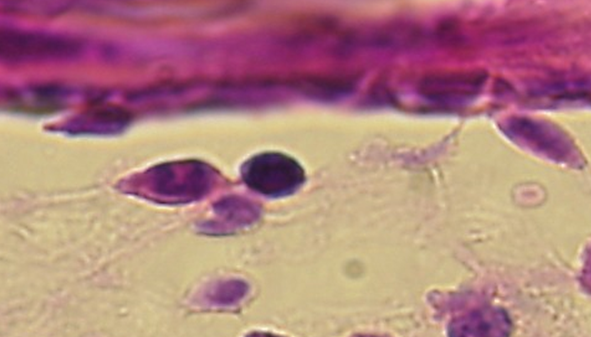

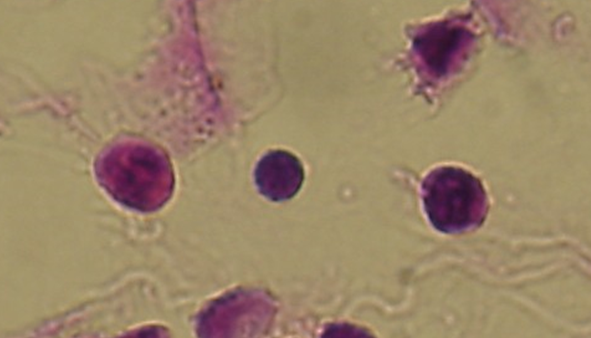


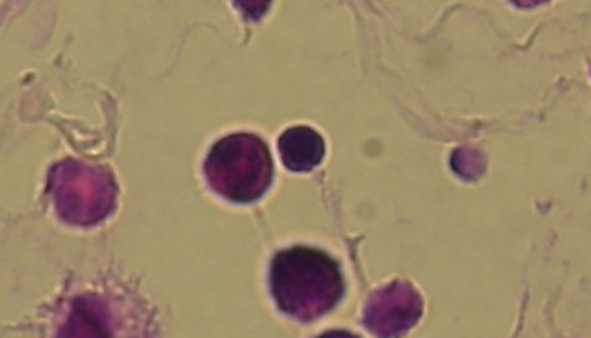


**Supplementary Figure 5.** The number of lymphocytes in lesional skin was counted. Mice were painted with vehicle or DNFB twice 14 days apart. The tissue was recovered 1 day or 5 day after 2^nd^ painting. Five mice were used for each data (n=5). Five to six fields (×600) per section were counted. Three sections were observed per mouse. Eighteen fields were examined in total. An arrow indicates a typical lymphocyte (×1000). Other immune cells are represented in these figures.

Lymphocyte is smaller than other immune cells. Lymphocyte has a large, dense, round nucleus, which accounts for most of the cell and thin cytoplasm (Anatomy Atlases).

**Supplementary Table 4.** Primer list

| gene | Sequence (5’→3’) |
| --- | --- |
| *IL-4* forward**^1^** | GAA TGT ACC AGG AGC CAT ATC |
| *IL-4* Reverse**^1^** | CTC AGT ACT ACG AGT AAT CCA |
| *IL-6* forward**^2^** | TGG AGT CAC AGA AGG AGT GGC TAAG |
| *IL-6* Reverse**^2^** | TCT GAC CAC AGT GAG GAA TGT CCA C |
| *IL-10* forward**^3^** | AGA AGC ATG GCC CTG AAA TCA AGG |
| *IL-10* Reverse**^3^** | CTT GTA GAC ACC TTG GTC TTG GAG |
| *TNF-α*forward**^4^** | CTG TAG CCC ACG TCG TAGC |
| *TNF-α*Reverse**^4^** | TTG AGA TCC ATG CCG TTG |
| *IL-1 β* forward**^5^** | CAACCAACAAGTGATATTCTCCATG |
| *IL-1 β*  Reverse**^5^** | GATCCACACTCTCCAGCTGCA |
| *IL-17a* forward**^5^** | TTTAACTCCCTTGGCGCAAAA |
| *IL-17a* Reverse**^5^** | CTTTCCCTCCGCATTGACAC |
| *IFN-γ* forward**^5^** | GGCCATCAGCAACAACATAAGCGT |
| *IFN- γ* Reverse**^5^** | TGGGTTGTTGACCTCAAACTTGGC |
| *filaggrin* forward**^5^** | ATGTCCGCTCTCCTGGAAAG |
| *filaggrin* Reverse**^5^** | TGGATTCTTCAAGACTGCCTGTA |
| *SPT5* forward**^6^** | GGT CCT ACT GAG CAT TGA TGG TGA G |
| *SPT5* Reverse**^6^** | TCA GGC TTC CAG GAG CTT CCC TAG G |

References

1. Choi J.H. et al., Food and Chemical Toxicology 56, 195-203 (2013).

2. Wang G. et al., J Immunol. 180, 8306-8316 (2008).

3. Terashima A. et al., J Exp Med. 205, 2727-2733 (2008).

4. Su S.J. et al., Hum Vaccin Immunother. 11, 915-921 (2015).

5. PrimerBank (https://pga.mgh.harvard.edu/primerbank/)

6. Stanlie A. et al., Plos Genetics 8, e1002675-e1002675 (2012).


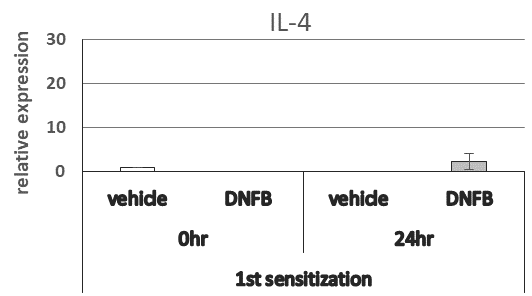

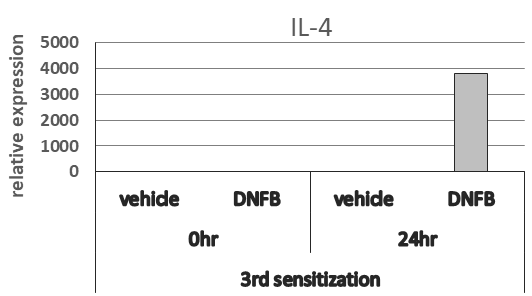


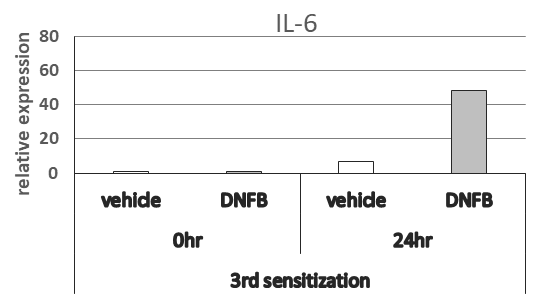

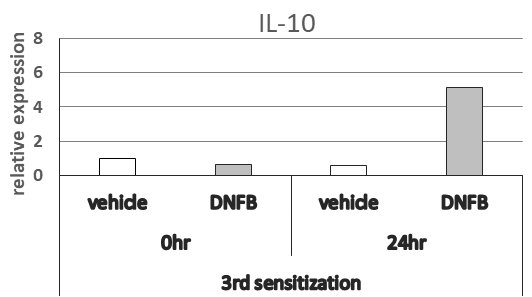


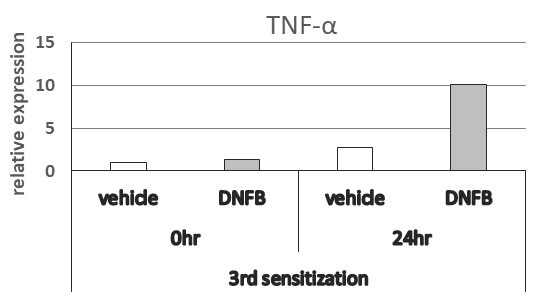


**Supplementary Figure 6.** Expression of inflammatory cytokines in the painted skin before and 24 h after the first and third painting session in Protocol 5. Total RNA was extracted from the dorsal skin of the Protocol 5-treated mice before (0 h) and 24 h after the first and third DNFB painting sessions. Quantitative PCR was performed to determine the mRNA expression of IL-4 of the first (n=3–4) and third painting (n=2) and IL-6, IL-10 and TNF-α of the third (n=2) painting. Mean and s.d. for IL-4 and mean for others are indicated.
